# Causal relationships between immune cells, plasma metabolites and lung adenocarcinoma: a two-step, two-sample Mendelian randomization study

**DOI:** 10.7150/jca.102760

**Published:** 2024-10-28

**Authors:** Weijun Zhou, Tian Yan, Zhuozheng Hu, Jiajun Wu, Yajie Zhou, Lei Xie, Jiao Yu, Wenxiong Zhang, Xinliang Liu

**Affiliations:** 1 Department of Thoracic Surgery, The Second Affiliated Hospital, Jiangxi Medical College, Nanchang University, Nanchang, 330006, China.; 2 Nursing Department, 82nd Army Group Hospital, Baoding, 071000, China.

**Keywords:** Mendelian randomization, Immune cells, Lung adenocarcinoma, Plasma metabolites

## Abstract

**Background:** Immune cells are crucial components of the immune system and significantly influence tumor progression. However, their direct involvement in lung adenocarcinoma (LUAD) and the potential mediation by plasma metabolites remain unclear. We performed a two-step, two-sample Mendelian randomization (MR) study to explore these connections between immune cells, plasma metabolites, and LUAD.

**Methods:** We collected data from the GWAS database and performed an MR study employing the inverse variance weighting (IVW) method. We calculated the total effect of immune cells on LUAD, the effect of immune cells on plasma metabolites, and the effect of plasma metabolites on LUAD. Additionally, we calculated the mediating effect and mediated proportion to explore the causal role of immune cells on LUAD and the mediating role of related metabolites in this association.

**Results:** Mendelian randomization analysis identified a causal relationship between 14 immune cell traits (Plasmacytoid DC %DC, Granulocyte %leukocyte, CCR2 on granulocyte, etc.) and LUAD, while LUAD showed no causal relationship with these 14 immune cell traits. Furthermore, 21 plasma metabolites (Gamma-glutamylmethionine levels, Malonylcarnitine levels, Linoleoyl ethanolamide levels, etc.) were suggestively associated with LUAD. Moreover, a causal relationship was identified between these plasma metabolites and 11 immune cell traits. Notably, mediator MR analysis identified 9 mediating pathways (CCR2 on granulocyte via 5alpha-androstan-3beta, 17alpha-diol disulfate, etc.). KEGG enrichment analysis revealed significant enrichment in the Valine, leucine and isoleucine biosynthesis.

**Conclusions:** Immune cells can affect the risk of LUAD through the above 9 pathways based on plasma metabolites which provide potential insights for constructing risk models for LUAD and identifying clinical biomarkers.

## Introduction

Lung adenocarcinoma (LUAD), which is the most common form of lung cancer, stands as one of the primary contributors to cancer-related mortality on a global scale. Its incidence has increased, especially among women and non-smokers 1-2. Despite advancements in diagnosis and treatment, LUAD has a poor prognosis and a low five-year survival rate 3. Current treatment strategies include surgery, radiotherapy, chemotherapy, and targeted therapy 4, but tumor heterogeneity and resistance lead to recurrence and metastasis, possibly due to unclear mechanisms underlying LUAD. Research indicates that immune cells in the tumor microenvironment play a dual role in both anti-tumor responses and tumor progression in LUAD 5.

Recent studies have discovered that immune cells exhibit both anti-tumor and tumor-promoting effects 5. In LUAD, the infiltration of immune cells such as T cells, NK cells, and macrophages is closely associated with patient prognosis 6. Studies have shown that the tumor-infiltrating lymphocytes (TILs) can predict clinical outcomes in LUAD patients 7. The impact of immune cells on cancer progression can be influenced by various factors, such as metabolites. These metabolites play a crucial mediating role in the interactions between tumor cells and immune cells, affecting tumor growth and immune evasion 8. Plasma metabolites offer a non-invasive approach to understanding the metabolic alterations associated with cancer and holds promise for identifying novel biomarkers and therapeutic targets 9.

Mendelian randomization (MR) uses genetic variants as instrumental variables to infer causal links between exposures and outcomes. This approach helps to minimize confounding factors and the risk of reverse causation 10, which provides more reliable evidence of causality compared to traditional observational studies 11. Recent studies have employed MR to explore the causal roles of various risk factors in cancer and to identify novel etiological factors and therapeutic targets 12. However, the application of MR to investigate the interplay between immune cells, Plasma metabolites, and LUAD is still in its early stages, presenting an opportunity for further research.

To investigate the causal relationship between immune cells, mediated by plasma metabolites, and LUAD, we used the Inverse Variance Weighted (IVW) method to identify immune cells and plasma metabolites causally related to LUAD. Subsequently, we calculated the mediating effect and mediated proportion among different groups based on their effect sizes to identify the mediating pathways involved.

## Materials and methods

### Design and data sources

**Figure 1:**
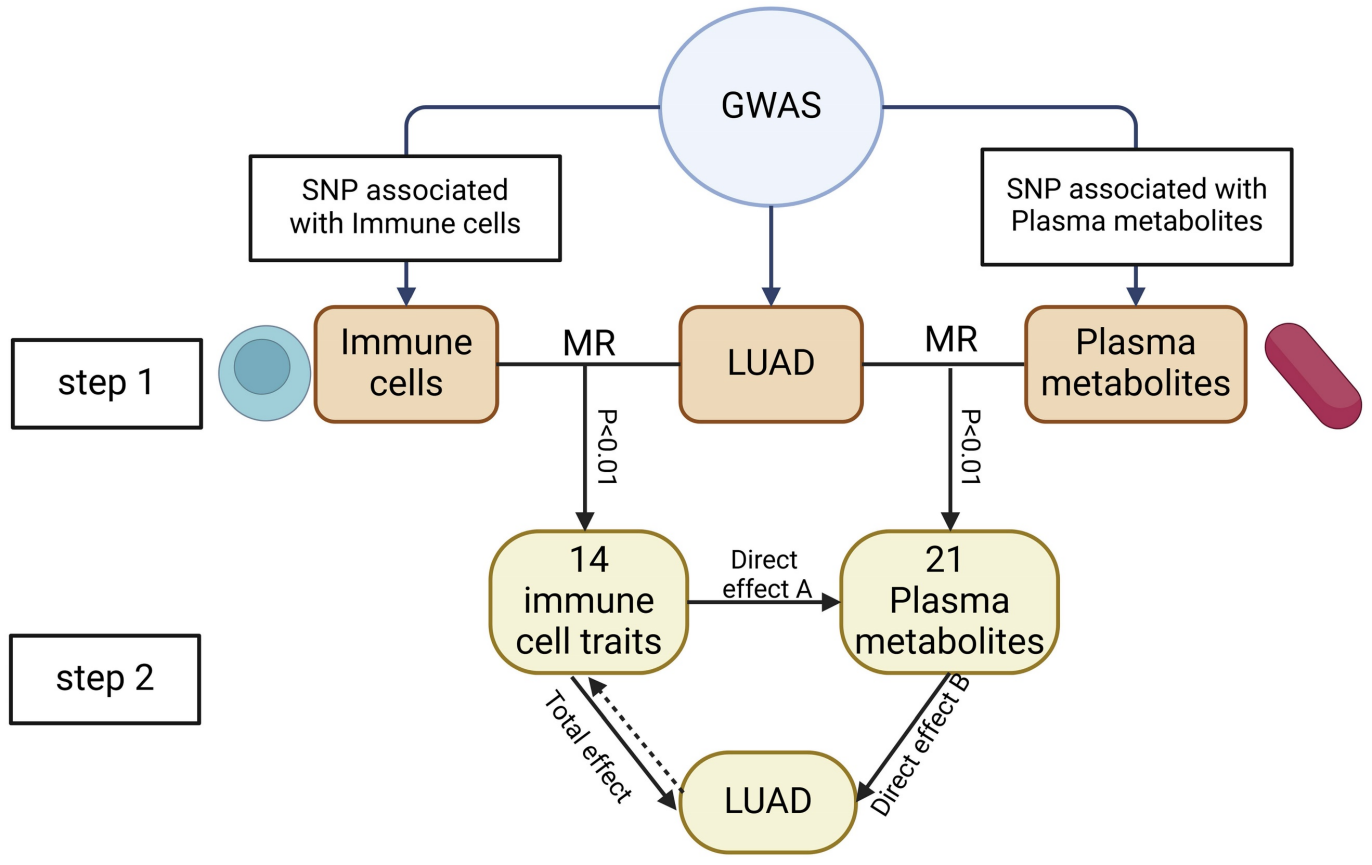
This study's design and flowchart. **Note:** Total effect: The causal role of immune cell traits on LUAD; Direct effect A: The causal role of immune cell traits on plasma metabolites; Direct effect B: The causal role of plasma metabolome on LUAD.

We conducted a two-step MR to determine the relationship between immune cell traits and the risk of LUA) and to assess whether plasma metabolites mediate this association. First, we evaluate the causal relationship of immune cell traits and plasma metabolites with LUAD, identifying immune cell traits and plasma metabolites associated with LUAD risk. The second step filtered the immune cell traits and plasma metabolites and assessed their causal relationship, and calculating the mediation proportion of each mediator on the impact of immune cell traits on LUAD. The study subjects should not overlap, meaning the SNPs representing exposure and outcome should come from different study sources. The study design is illustrated in **Figure 1**.

Data on 731 immune cell traits (Ebi-a-GCST0001391 to Ebi-a-GCST0002121) were obtained from the GWAS database (https://gwas.mrcieu.ac.uk/) 13. These 731 immune cell traits included: B cells, CDC, mature T cells, monocytes, myeloid cells, TBNK (B cells, natural killer cells, T cells), and Treg groups. Data on 1,091 plasma metabolites and 309 metabolite ratios were acquired from the GWAS database (Accession numbers for European GWASs: GCST90199621-90201020) 14. For this study, the GWAS summary statistics for LUAD (ieu-a-965) were obtained from the ILCCO consortium. Initiated in 2004, the ILCCO (International Lung Cancer Consortium) involves hundreds of thousands of samples from multiple countries and regions worldwide. It aims to integrate genetic and environmental data from different cohorts to study the mechanisms of lung cancer pathogenesis, thereby advancing prevention, diagnosis, and treatment of lung cancer. The data include 8,881,354 SNPs from 18,336 samples of European ancestry.

### Selection of genetic Instrumental Variables (IVs)

The selection criteria for exposure and outcome SNPs, including immune cell traits, plasma metabolites, and LUAD, were set at p < 1e-5. All genetic variants were grouped together using a clumping threshold of R²<0.001 within a clumping distance of 10,000 kb, ensuring that only variants with minimal linkage disequilibrium were included 15. The F-statistic (β divided by the square of the standard error) was used to filter SNPs, with a cutoff value of 10. SNPs with an F-statistic less than 10 were considered to have significant weak instrument bias and were excluded from the study to enhance the validity of our results 16.

### Statistical analysis

All statistical analyses were performed using R version 4.3.3 (https://www.r-project.org). The "TwoSampleMR" package, "VariantAnnotation" package, and "ieugwasr" package were used for two-sample MR analyses. Among the five MR methods ("MR Egger", "weighted median", "IVW", "simple mode" and "weighted mode") 17-20, IVW was the primary method for causal estimation because it is the most precise and robust method. A p-value < 0.001 was used to identify significant associations between exposures (immune cell traits, plasma metabolites) and the outcome (LUAD) for further evaluation. Cochran's Q statistic, which was used to assess heterogeneity based on the IVW and MR Egger methods. The MR-Egger intercept test and the MR-PRESSO method, via the "MR-PRESSO" package, were used to detect pleiotropy and correct for horizontal pleiotropy by removing outliers. A leave-one-out analysis was conducted to explore the impact of potential outlier genetic variants. Additionally, we conducted KEGG and SMPDB enrichment analyses using MetaboAnalyst 5.0 (https://www.metaboanalyst.ca/), an online platform designed for comprehensive metabolomic data analysis.

### Mediation analysis

Mediation analysis could assess the role of a third variable in the mechanism by which the exposure factor induces the outcome factor. Through mediation MR, we identified pathways from immune cell traits to plasma metabolites to LUAD, helping to elucidate the potential mechanisms by which immune cell traits might promote LUAD. First, we conducted mediation analysis to evaluate the possible link between MR-identified immune cell traits and plasma metabolites. Subsequently, we assessed the "indirect" effect of immune cell traits on LUAD via plasma metabolites using a two-step MR process. The calculation formula were as follows: The mediated proportion = β(Mediated effect) / β(total effect); β(Mediated effect) = β(Direct effect A)×β(Direct effect B); Total effect: The causal role of immune cell traits on LUAD; Direct effect A: The causal role of immune cell traits on plasma metabolites; Direct effect B: The causal role of plasma metabolome on LUAD 21.

## Results

### The overall impact of immune cells on LUAD

**Figure 2:**
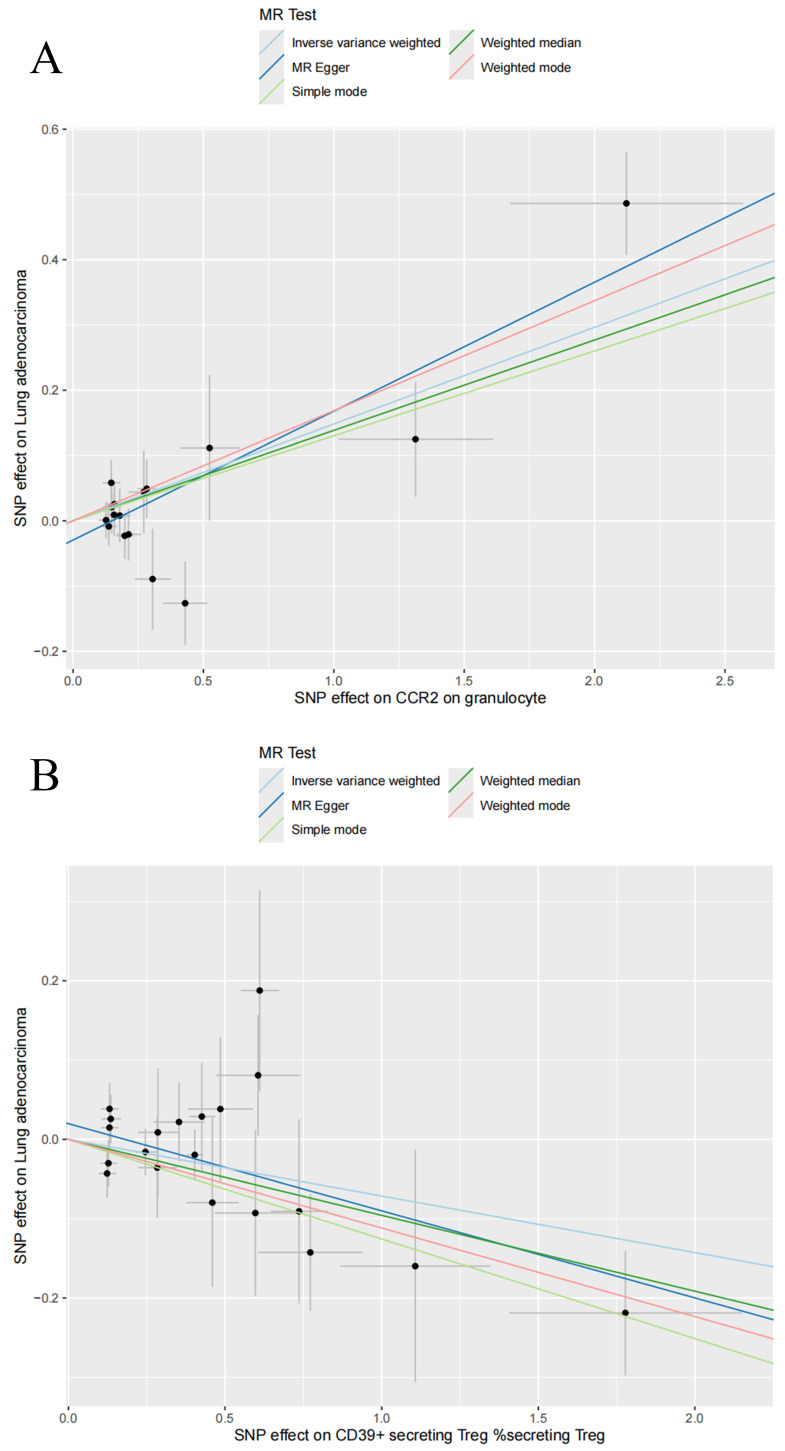
Scatter plots for the causal association between immune cell traits and lung adenocarcinoma. Scatter plots of CCR2 on granulocyte (A) and CD39+ secreting Treg %secreting Treg (B).

In this study, 18,621, 34,844, and 10,045 SNPs were selected as IVs for 731 immune cell traits, 1,400 metabolites, and LUAD. Two-sample MR analysis demonstrated a causal relationship between 14 immune cell traits and LUAD (**Table 1**). Using the IVW method, it was shown that an increase in the following four immune cell traits is associated with an increased risk of LUAD: Plasmacytoid DC %DC (OR: 1.10 [1.03, 1.18], P = 0.028), Granulocyte %leukocyte (OR: 1.14 [1.04, 1.26], P = 0.0062), CCR2 on granulocyte (OR: 1.16 [1.08, 1.24], P = 2.96E-05) and CD25 on CD45RA+ CD4 not Treg (OR: 1.12 [1.05, 1.21], P = 0.0013). The remaining 10 immune cell traits were associated with a reduced risk of LUAD, including CD39+ secreting Treg %secreting Treg (OR: 0.93 [0.88, 0.98], P = 0.0087), Naive CD8br %CD8br (OR: 0.91 [0.85, 0.98], P = 0.0089), T cell %leukocyte (OR: 0.91 [0.85, 0.97], P = 0.0043), CD28+ CD45RA+ CD8dim %T cell (OR: 0.96 [0.94, 0.99], P = 0.0061), CD28+ CD45RA+ CD8dim AC (OR: 0.97 [0.95, 0.99], P = 0.0034), CD28- CD127- CD25++ CD8br %T cell (OR: 0.84 [0.75, 0.95], P = 0.0042), CD19 on IgD+ CD38br (OR: 0.83 [0.75, 0.92], P = 0.0006), CD19 on IgD- CD27- (OR: 0.85 [0.78, 0.93], P = 0.0003), CD25 on resting Treg (OR: 0.89 [0.82, 0.96], P = 0.0022), and CD25 on CD4+ (OR: 0.88 [0.81, 0.96], P = 0.0023). At the same time, we visualized the causal relationship between immune cell traits and LUAD through scatter plots (**Figure 2, Figure S1**). Heterogeneity tests and pleiotropy tests indicated that only the immune cell trait CD28+ CD45RA+ CD8dim AC showed heterogeneity, which may be due to different study methods. Furthermore, we did not observe any horizontal pleiotropy (**Table S1, Table S2**). The leave-one-out analysis indicated that removing any specific SNP did not alter the causal estimates (**Figure S2**).

### Reverse MR analysis

Through previous analyses, we demonstrated a causal relationship between 14 immune cell traits and LUAD. To investigate the potential causal relationship of LUAD on these 14 immune cell traits, we subsequently performed reverse MR analysis. The results indicated that LUAD has no significant causal relationship with any of the 14 immune cell traits (**Table S3**).

### The impact of plasma metabolites on LUAD

**Figure 3:**
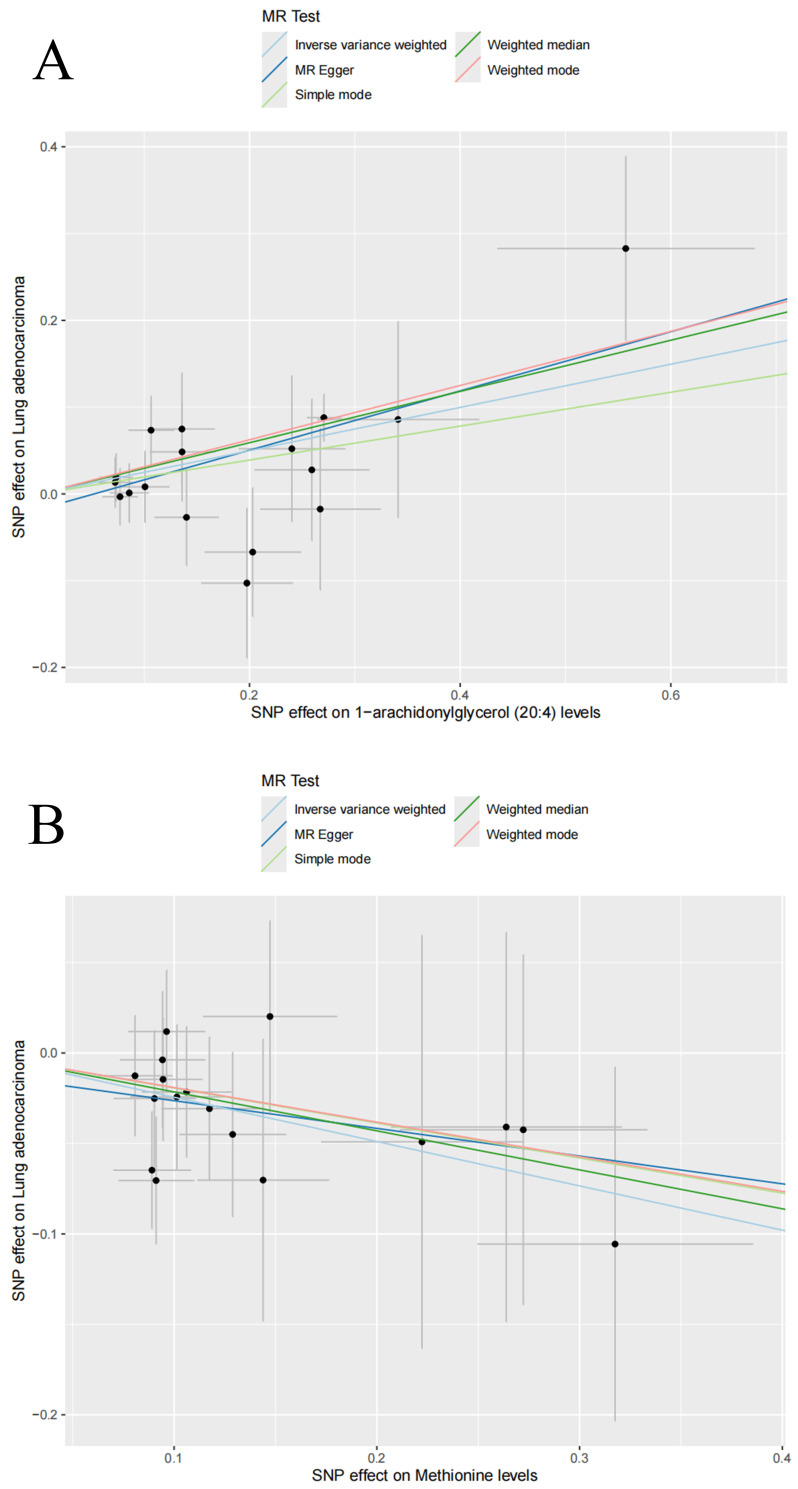
Scatter plots for the causal association between plasma metabolites and lung adenocarcinoma. Scatter plots of 1-arachidonylglycerol (20:4) levels (A) and Methionine levels (B).

**Figure 4:**
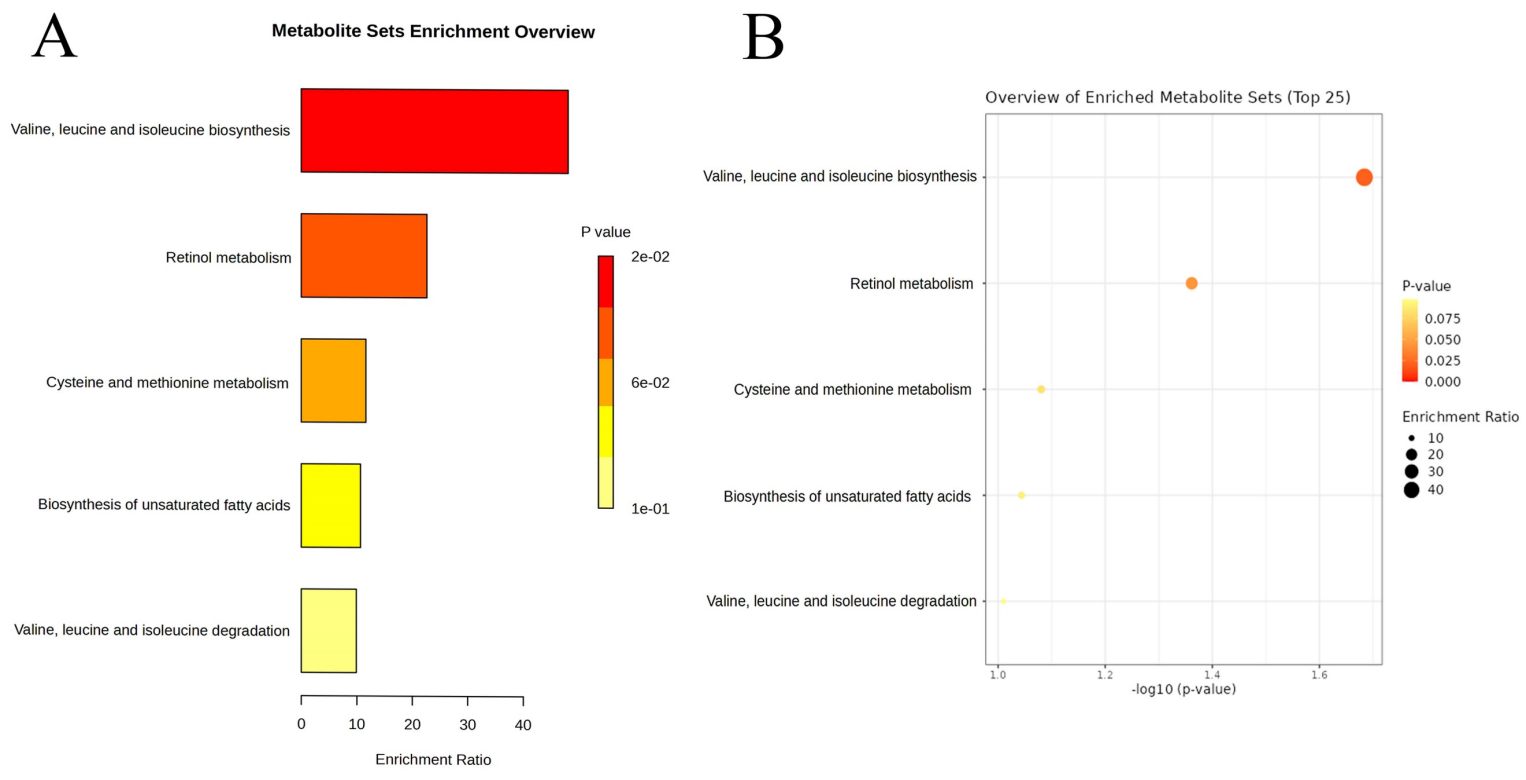
Enrichment analysis results of the causal plasma metabolites of lung adenocarcinoma based on the Kyoto Encyclopedia of Genes and Genomes database: Bar chart (A) and Dot Plot (B).

Among the 1,400 metabolites analyzed, the IVW method identified 21 plasma metabolites that influence the risk of LUAD (**Table 2**). A dynamic ring heatmap was utilized to compare changes in beta, se, or, and P values of plasma metabolites across different groups (**Figure S3**). 11 plasma metabolites were associated with a decreased risk of LUAD, including Gamma-glutamylmethionine levels, Malonylcarnitine levels, Linoleoyl ethanolamide levels, 1-stearoyl-2-linoleoyl-GPC (18:0/18:2) levels, Dibutyl sulfosuccinate levels, 1-palmitoyl-2-linoleoyl-GPC (16:0/18:2) levels, 1-palmitoyl-2-linoleoyl-GPI (16:0/18:2) levels, Methionine levels, Retinol (Vitamin A) to linoleoyl-arachidonoyl-glycerol (18:2 to 20:4) ratio and Cholesterol to linoleoyl-arachidonoyl-glycerol (18:2 to 20:4) ratio. The remaining ten metabolites were associated with an increased risk of LUAD, including 4-methyl-2-oxopentanoate levels, 1-arachidonylglycerol (20:4) levels, 1-arachidonoyl-GPC (20:4n6) levels, 1-arachidonoyl-GPE (20:4n6) levels, 5alpha-androstan-3beta, 17alpha-diol disulfate levels, Indole-3-carboxylate levels, 2-aminophenol sulfate levels, 1H-indole-7-acetic acid levels, 3-hydroxyhexanoate levels, and Eicosapentaenoate (EPA; 20:5n3) levels. Scatter plots showed consistent results (**Figure 3, Figure S4**). Heterogeneity and pleiotropy tests indicated that only Dibutyl sulfosuccinate levels exhibited heterogeneity, which may be due to different study methods. Through horizontal pleiotropy study, no horizontal pleiotropy was found in MR analysis (**Table S4, Table S5**). The leave-one-out analysis demonstrated that removing any SNP did not alter the causal estimates (**Figure S5**). In addition, we conducted KEGG and SMPDB enrichment analyses on a set of 21 plasma metabolites. KEGG enrichment analysis revealed significant enrichment in valine, leucine, and isoleucine biosynthesis (**Figure 4**). Meanwhile, SMPDB enrichment analysis revealed significant enrichment in alpha-linolenic acid and linoleic acid metabolism (**Figure S6**).

### Impact of immune cells on metabolites

Previously, we identified 14 immune cell traits and 21 plasma metabolites that have a causal relationship with LUAD. Next, we examined the causal effects of the 14 immune cell traits on the 21 plasma metabolites. MR analysis revealed 11 causal relationships, including CD39+ secreting Treg %secreting Treg and 1-arachidonylglycerol (20:4) levels (OR: 0.973 [0.948, 0.998], P = 0.036), CD39+ secreting Treg %secreting Treg and 1-stearoyl-2-linoleoyl-GPC (18:0/18:2) levels (OR: 0.974 [0.950, 0.999], P = 0.042), T cell %leukocyte and Linoleoyl ethanolamide levels (OR: 1.053 [1.011, 1.096], P = 0.014), CD28+ CD45RA+ CD8dim %T cell and Indole-3-carboxylate levels (OR: 1.015 [1.000, 1.030], P = 0.046), CD28+ CD45RA+ CD8dim AC and Indole-3-carboxylate levels (OR: 1.014 [1.005, 1.024], P = 0.003), CD28- CD127- CD25++ CD8br %T cell and 3-hydroxyhexanoate levels (OR: 1.049 [1.002, 1.097], P = 0.039), CD28- CD127- CD25++ CD8br %T cell and Methionine levels (OR: 0.943 [0.897, 0.992], P = 0.023), CD19 on IgD+ CD38br and Cholesterol to linoleoyl-arachidonoyl-glycerol (18:2) ratio (OR: 0.947 [0.899, 0.998], P = 0.043), CD25 on CD45RA+ CD4 not Treg and Linoleoyl ethanolamide levels (OR: 1.034 [1.000, 1.069], P = 0.047), CD25 on CD4+ and Methionine levels (OR: 1.035 [1.004, 1.068], P = 0.028), and CCR2 on granulocyte and 5alpha-androstan-3beta, 17alpha-diol disulfate levels (OR: 1.042 [1.003, 1.082], P = 0.036). In this MR analysis, heterogeneity was not found, nor was horizontal pleiotropy. The leave-one-out analysis offered further confirmation that the observed causal associations are robust and not influenced by any single SNP.

### Mediated effect of immune cell traits on LUAD

**Figure 5:**
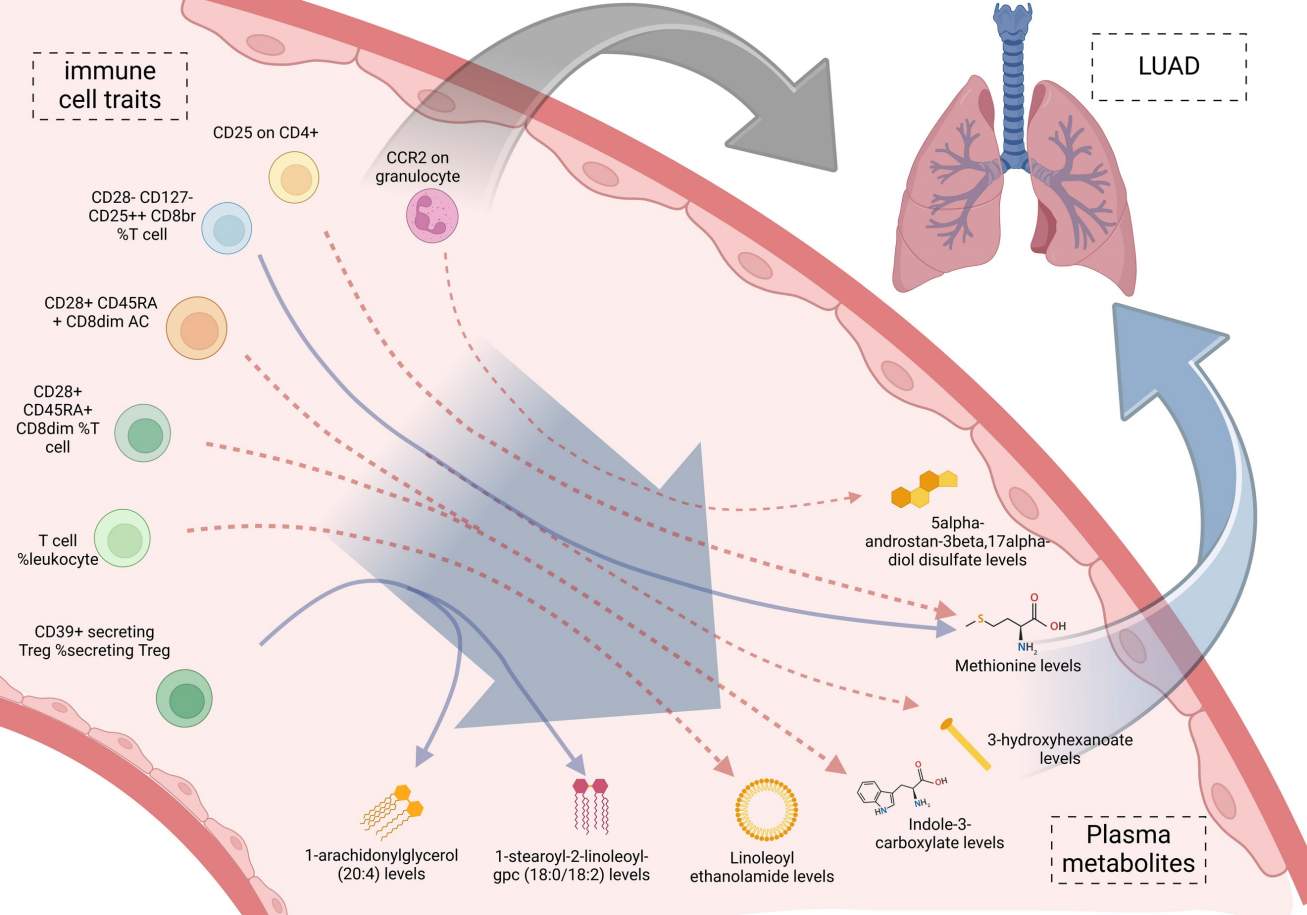
Mediation effect relationship of immune cell traits and plasma metabolites on lung adenocarcinoma.

**Figure 6:**
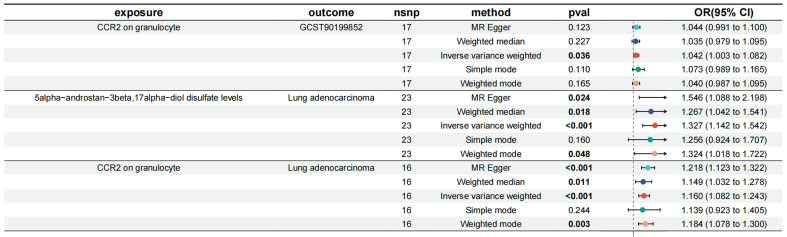
Forest plot of CCR2 on granulocyte via 5alpha-androstan-3beta, 17alpha-diol disulfate.

We incorporated the 11 immune cell traits and plasma metabolites identified as having causal relationships into a mediation analysis to determine the potential pathways through which immune cell traits and metabolites influence LUAD. The mediation analysis identified a total of 9 distinct mediation pathways, providing insights into the mechanisms at play: 9.71% of the effect of CD39+ secreting Treg %secreting Treg on LUAD is mediated by 1-arachidonylglycerol (20:4) levels; 9.00% of the effect of CD39+ secreting Treg %secreting Treg on LUAD is mediated by 1-stearoyl-2-linoleoyl-GPC (18:0/18:2) levels; 11.60% of the effect of T cell %leukocyte on LUAD is mediated by Linoleoyl ethanolamide levels; 9.59% of the effect of CD28+ CD45RA+ CD8dim %T cell on LUAD is mediated by Indole-3-carboxylate levels; 10.00% of the effect of CD28+ CD45RA+ CD8dim AC on LUAD is mediated by Indole-3-carboxylate levels; 7.34% of the effect of CD28- CD127- CD25++ CD8br %T cell on LUAD is mediated by 3-hydroxyhexanoate levels; 8.39% of the effect of CD28- CD127- CD25++ CD8br %T cell on LUAD is mediated by Methionine levels; 6.77% of the effect of CD25 on CD4+ on LUAD is mediated by Methionine levels; and 7.82% of the effect of CCR2 on granulocyte on LUAD is mediated by 5alpha-androstan-3beta, 17alpha-diol disulfate levels (**Table 3, Figure 5**). Specifically, we identified that CCR2 on granulocytes has a direct effect on LUAD with an OR (Odds Ratio) of 1.160, indicating an increased risk of LUAD. Additionally, CCR2 on granulocytes influences the levels of 5alpha-androstan-3beta, 17alpha-diol disulfate, with an OR of 1.042, suggesting a slight increase in this metabolite. The metabolite itself has an OR of 1.327 in relation to LUAD, further contributing to the overall increased risk. Together, these findings suggest that CCR2 on granulocytes promotes LUAD progression through its mediation by 5alpha-androstan-3beta, 17alpha-diol disulfate, and both serve as risk factors in this pathway. And then the specific mediating effects of each plasma metabolite were reflected by forest plots (**Figure 6, Figure S7**).

## Discussion

Even with notable advancements in the early detection and treatment of LUAD, the overall outlook is still grim, as evidenced by the persistently low five-year survival rate. This highlights the necessity of identifying new etiological factors and therapeutic targets. Innovative approaches are crucial for discovering new therapeutic pathways. MR analysis mitigates confounding and reverse causation, presenting a robust approach to deduce causal links between risk factors and outcomes. Several studies have utilized MR to investigate how metabolites mediate the progression of cancer.

For example, Zeng *et al.* explored the causal impact of gut microbiota and plasma metabolome on lung cancer and the heterogeneity across subtypes 22, Chen *et al.* investigated the causal links between gut microbiota, immune cells, and lung cancer 23. These studies underscore the potential of MR in uncovering complex biological interactions and identifying new therapeutic targets. In our study, we conducted a MR analysis to explore the causal relationships between immune cells, plasma metabolites, and LUAD. Using the inverse-variance weighted (IVW) method, we assessed the total causal effect of immune cells on LUAD and further analyzed the causal relationships between immune cells and metabolites as well as between metabolites and LUAD, identifying 9 mediation pathways. This comprehensive analysis provides new insights into the complex relationships between immune cells and LUAD and offers potential directions for future therapeutic strategies.

In the LUAD tumor microenvironment, immune cells play a vital role, influencing the progression of tumor and patient prognosis. Our MR analysis identified 14 immune cell traits with causal relationships to LUAD. The lack of a significant causal relationship between LUAD and the 14 immune cell traits in the reverse MR analysis suggests that these immune traits may function as predisposing factors for LUAD rather than being influenced by the disease itself. Specifically, four immune cell traits were associated with increased LUAD risk, including plasmacytoid dendritic cells, granulocytes, non-regulatory CD25+ CD45RA+ CD4+ T cells, and CCR2 on granulocytes. The accumulation of plasmacytoid dendritic cells has been found to promote cancer immune evasion and tumor growth 24. Granulocytes, particularly neutrophils, have been shown to support tumor progression through promoting inflammation and immune suppression in various cancers 25. Non-regulatory CD25+ CD45RA+ CD4+ T cells and CCR2 on granulocytes may boost the immunosuppressive characteristics of the tumor environment, thereby promoting LUAD progression 26. Conversely, 10 immune cell traits were associated with reduced LUAD risk. For example, CD39+ secreting regulatory T cells play a significant role in immunosuppression; A reduction in these cells could indicate a decrease in immune suppression within the tumor microenvironment, potentially leading to an inhibition of tumor growth 27. An increase in the proportion of naive CD8 high-expressing cells and T cells is associated with enhanced anti-tumor immune responses, as previous studies have shown that these cells can inhibit LUAD by directly killing tumor cells and supporting the anti-tumor functions of other immune cells 28. These findings indicate that some immune cell traits can drive tumor progression and be targeted for therapy, while others can inhibit tumor development by enhancing immune responses.

The significance of plasma metabolites in LUAD is gaining increased recognition. Our findings revealed that 21 plasma metabolites have significant causal links to LUAD. Among them, metabolites such as gamma-glutamylmethionine, malonylcarnitine, linoleoyl ethanolamide (LEA), 1-stearoyl-2-linoleoyl-GPC (18:0/18:2), and dibutyl sulfosuccinate were associated with increased LUAD risk 8-9. For example, gamma-glutamylmethionine may promote tumor development by affecting cellular oxidative stress levels 29. LEA, an endogenous fatty acid ethanolamide, has been shown to regulate immune cell function and metabolic status in various tumors 9. LEA's involvement in immune regulation suggests that it could be used to monitor immune system alterations before clinical symptoms of LUAD appear, offering a window for early intervention. Additionally, other metabolites such as 1-arachidonylglycerol (20:4) and 1-arachidonoyl-GPC (20:4n6) were found to be associated with reduced LUAD risk, possibly due to their complex roles in cellular signaling and metabolic regulation 30.

To further investigate how metabolites mediate the relationship between immune cell traits and LUAD, we performed mediation analysis. The results revealed 9 significant mediation pathways between immune cell traits and LUAD. For example, 9.71% of the effect of CD39+ secreting regulatory T cells on LUAD was mediated by 1-arachidonylglycerol (20:4), and 11.60% of the effect of T cell %leukocyte on LUAD was mediated by LEA. LEA's role especially caught our interest. It has demonstrated anti-inflammatory and immune-regulating effects in various tumors. Its increased levels are associated with an increase in T cell %leukocyte, and LEA exerts a protective effect against LUAD, reducing the risk of LUAD development 9,30. What's more, KEGG enrichment analysis highlighted significant enrichment in the biosynthesis of valine, leucine, and isoleucine, while SMPDB enrichment analysis showed significant enrichment in alpha-linolenic acid and linoleic acid metabolism. These three branched-chain amino acids (BCAAs) are vital for protein synthesis, energy metabolism, and immune regulation 31. Previous research links disturbances in BCAA metabolism to metabolic disorders like obesity and diabetes 32. Alpha-linolenic acid and linoleic acid are essential polyunsaturated fatty acids (PUFAs) that play crucial roles in various physiological processes. Studies suggest that abnormal PUFA metabolism may contribute to various chronic diseases, including cardiovascular conditions and cancer 33,34.

We present several innovations and limitations. First, we systematically evaluated the causal relationships between immune cell traits, metabolites, and LUAD using MR for the first time, revealing multiple metabolites that mediate this process. This provides a new perspective for understanding the complex interactions between immune cells and metabolites in lung adenocarcinoma and offers potential targets for future therapeutic strategies. These findings could also inform the selection of patients for clinical trials, ensuring that therapies are tailored to those most likely to benefit based on their metabolic and immune profiles. However, the study also has some limitations. Although MR analysis has advantages in controlling for confounding factors, the results may still be influenced by the selection of instrumental variables and sample size. Additionally, while we have identified multiple significant causal relationships, further experimental validation is needed to confirm these findings and establish their clinical relevance.

## Conclusion

The risk of LUAD have a causal relationship with the 14 immune cell traits (CCR2 on granulocyte, etc.). Additionally, 21 plasma metabolites (Linoleoyl ethanolamide levels, etc.) also influence the risk of LUAD. The impact of immune cells on LUAD is mediated through 9 pathways (CCR2 on granulocyte via 5alpha-androstan-3beta, 17alpha-diol disulfate, etc.) involving plasma metabolites. These insights offer potential avenues for constructing risk models for LUAD and identifying clinical biomarkers.

## Supplementary Material

Supplementary figures and tables.

Supplementary figures only (higher quality).

## Figures and Tables

**Table 1:** Causal associations between immune cells and lung adenocarcinoma by using the inverse-variance weighted method.

Exposure	Nsnp	B	Se	OR	OR_LCI95	OR_UCI95	P-value
Plasmacytoid DC %DC	21	0.10	0.03	1.10	1.03	1.18	0.0028
CD39+ secreting Treg %secreting Treg	20	-0.07	0.03	0.93	0.88	0.98	0.0087
Naive CD8br %CD8br	24	-0.09	0.03	0.91	0.85	0.98	0.0089
T cell %leukocyte	15	-0.10	0.03	0.91	0.85	0.97	0.0043
Granulocyte %leukocyte	21	0.13	0.05	1.14	1.04	1.26	0.0062
CD28+ CD45RA+ CD8dim %T cell	33	-0.04	0.01	0.96	0.94	0.99	0.0061
CD28+ CD45RA+ CD8dim AC	35	-0.03	0.01	0.97	0.95	0.99	0.0034
CD28- CD127- CD25++ CD8br %T cell	14	-0.17	0.06	0.84	0.75	0.95	0.0042
CD19 on IgD+ CD38br	13	-0.18	0.05	0.83	0.75	0.92	0.0006
CD19 on IgD- CD27-	22	-0.16	0.04	0.85	0.78	0.93	0.0003
CD25 on CD45RA+ CD4 not Treg	20	0.12	0.04	1.12	1.05	1.21	0.0013
CD25 on resting Treg	14	-0.12	0.04	0.89	0.82	0.96	0.0022
CD25 on CD4+	12	-0.13	0.04	0.88	0.81	0.96	0.0023
CCR2 on granulocyte	16	0.15	0.04	1.16	1.08	1.24	2.96E-05

**Abbreviations:** B: Beta-value; IVW: Inverse-variance weighted; LCI: Lower confidence interval; LUAD: Lung adenocarcinoma; Nsnp: number of SNPs; OR: Odds ratio; Snp: Single nucleotide polymorphism; Se: Standard error; UCI: Upper confidence interval.

**Table 2:** Causal associations between plasma metabolites and lung adenocarcinoma by using the inverse-variance weighted method.

Exposure	Nsnp	B	Se	OR	OR_LCI95	OR_UCI95	P-value
4-methyl-2-oxopentanoate levels	12	0.32	0.11	1.38	1.11	1.71	3.45E-03
Gamma-glutamylmethionine levels	24	-0.20	0.08	0.81	0.70	0.95	9.63E-03
1-arachidonylglycerol (20:4) levels	17	0.25	0.07	1.28	1.13	1.46	1.44E-04
Malonylcarnitine levels	19	-0.25	0.09	0.78	0.65	0.93	4.87E-03
1-arachidonoyl-gpc (20:4n6) levels	23	0.12	0.04	1.13	1.04	1.22	3.34E-03
1-arachidonoyl-GPE (20:4n6) levels	29	0.13	0.05	1.14	1.04	1.25	4.55E-03
5alpha-androstan-3beta,17alpha-diol disulfate levels	23	0.28	0.08	1.33	1.14	1.54	2.21E-04
Indole-3-carboxylate levels	14	0.24	0.08	1.27	1.09	1.48	2.62E-03
2-aminophenol sulfate levels	25	0.21	0.08	1.23	1.05	1.44	8.79E-03
1H-indole-7-acetic acid levels	24	0.25	0.07	1.28	1.12	1.46	3.91E-04
3-hydroxyhexanoate levels	12	0.26	0.10	1.30	1.07	1.57	7.11E-03
Linoleoyl ethanolamide levels	20	-0.22	0.08	0.80	0.68	0.94	6.76E-03
1-stearoyl-2-linoleoyl-gpc (18:0/18:2) levels	18	-0.25	0.09	0.78	0.65	0.94	8.04E-03
Dibutyl sulfosuccinate levels	28	-0.29	0.10	0.75	0.62	0.91	3.49E-03
1-palmitoyl-2-linoleoyl-gpc (16:0/18:2) levels	27	-0.21	0.06	0.81	0.73	0.91	3.67E-04
1-palmitoyl-2-linoleoyl-GPI (16:0/18:2) levels	20	-0.22	0.07	0.80	0.69	0.93	2.61E-03
Eicosapentaenoate (EPA; 20:5n3) levels	22	0.23	0.08	1.26	1.08	1.47	4.04E-03
Methionine levels	17	-0.24	0.09	0.78	0.65	0.94	7.45E-03
Retinol (Vitamin A) to linoleoyl-arachidonoyl-glycerol (18:2 to 20:4) 1 ratio	9	-0.26	0.10	0.77	0.64	0.94	8.47E-03
Retinol (Vitamin A) to linoleoyl-arachidonoyl-glycerol (18:2 to 20:4) 2 ratio	12	-0.24	0.07	0.79	0.69	0.90	4.00E-04
Cholesterol to linoleoyl-arachidonoyl-glycerol (18:2 to 20:4) 1 ratio	16	-0.22	0.07	0.81	0.70	0.93	3.63E-03

**Abbreviations:** B: Beta-value; IVW: Inverse-variance weighted; LCI: Lower confidence interval; LUAD: Lung adenocarcinoma; Nsnp: number of SNPs; OR: Odds ratio; Snp: Single nucleotide polymorphism; Se: Standard error; UCI: Upper confidence interval. Note: 1 The data comes from the GCST90200907 dataset; 2 The data comes from the GCST90200908 dataset.

**Table 3:** Mediation Mendelian randomization analysis of the causal relationship between immune cell traits, plasma metabolites and lung adenocarcinoma.

Immune cells	Plasma metabolites	ME	MP	P-value
CD39+ secreting Treg %secreting Treg	1-arachidonylglycerol (20:4) levels	-0.00693	9.71%	0.037
CD39+ secreting Treg %secreting Treg	1-stearoyl-2-linoleoyl-gpc (18:0/18:2) levels	0.00642	-9.00%	0.043
T cell %leukocyte	Linoleoyl ethanolamide levels	-0.0114	11.60%	0.016
CD28+ CD45RA+ CD8dim %T cell	Indole-3-carboxylate levels	0.00354	-9.59%	0.047
CD28+ CD45RA+ CD8dim AC	Indole-3-carboxylate levels	0.00336	-10.00%	0.004
CD28- CD127- CD25++ CD8br %T cell	3-hydroxyhexanoate levels	0.0125	-7.34%	0.042
CD28- CD127- CD25++ CD8br %T cell	Methionine levels	0.0143	-8.39%	0.027
CD25 on CD4+	Methionine levels	-0.00851	6.77%	0.030
CCR2 on granulocyte	5alpha-androstan-3beta,17alpha-diol disulfate levels	0.0116	7.82%	0.037

**Abbreviations:** LUAD: Lung adenocarcinoma; ME: Mediated effect; MP: Mediated proportion.

## Abbreviations

BCAAs: Branched-chain amino acids
CI: Confidence interval
CCR2: C-C Motif Chemokine Receptor 2
DC: Dendritic cell
EPA: Eicosapentaenoate
GWAS: Genome-wide association study
ILCCO: International Lung Cancer Consortium
IVs: Instrumental Variables
IVW: Inverse variance weighting
KEGG: Kyoto Encyclopedia of Genes and Genomes
LEA: Linoleoyl ethanolamide
LUAD: Lung adenocarcinoma
MR: Mendelian randomization
NK cell: Natural killer cell
OR: Odds ratio
PD-1: Programmed death 1
PD-L1: Programmed death ligand 1
PUFAs: Polyunsaturated fatty acids
SNP: Single nucleotide polymorphism
SMPDB: Small Molecule Pathway Database
TILs: Tumor-infiltrating lymphocytes
