# Supplementary material for: Causal relationships between immune cells, plasma metabolites and lung adenocarcinoma: a two-step, two-sample Mendelian randomization study
Source: J Cancer. 2024 Oct 28;15(20):6698–709. doi: 10.7150/jca.102760 (PMC11632987; doi:10.7150/jca.102760)
Supplement: Supplementary file 1 — Supplementary figures and tables. [file jcav15p6698s1.pdf]

Supplementary Data

Figure S1 Scatter plots for the causal association between immune cell traits and lung adenocarcinoma. Scatter plots of T cell %leukocyte (A), Granulocyte %leukocyte (B), CD28+ CD45RA+ CD8dim %T cell (C), CD28+ CD45RA+ CD8dim AC (D), CD28- CD127- CD25++ CD8br %T cell (E), CD19 on IgD+ CD38br (F), CD19 on IgD- CD27- (G), CD25 on CD45RA+ CD4 not Treg (H), CD25 on resting Treg (I), CD25 on CD4+ (J), CCR2 on granulocyte (K), Plasmacytoid DC %DC (L), CD39+ secreting Treg %secreting Treg (M) and Naive CD8br %CD8br (N).

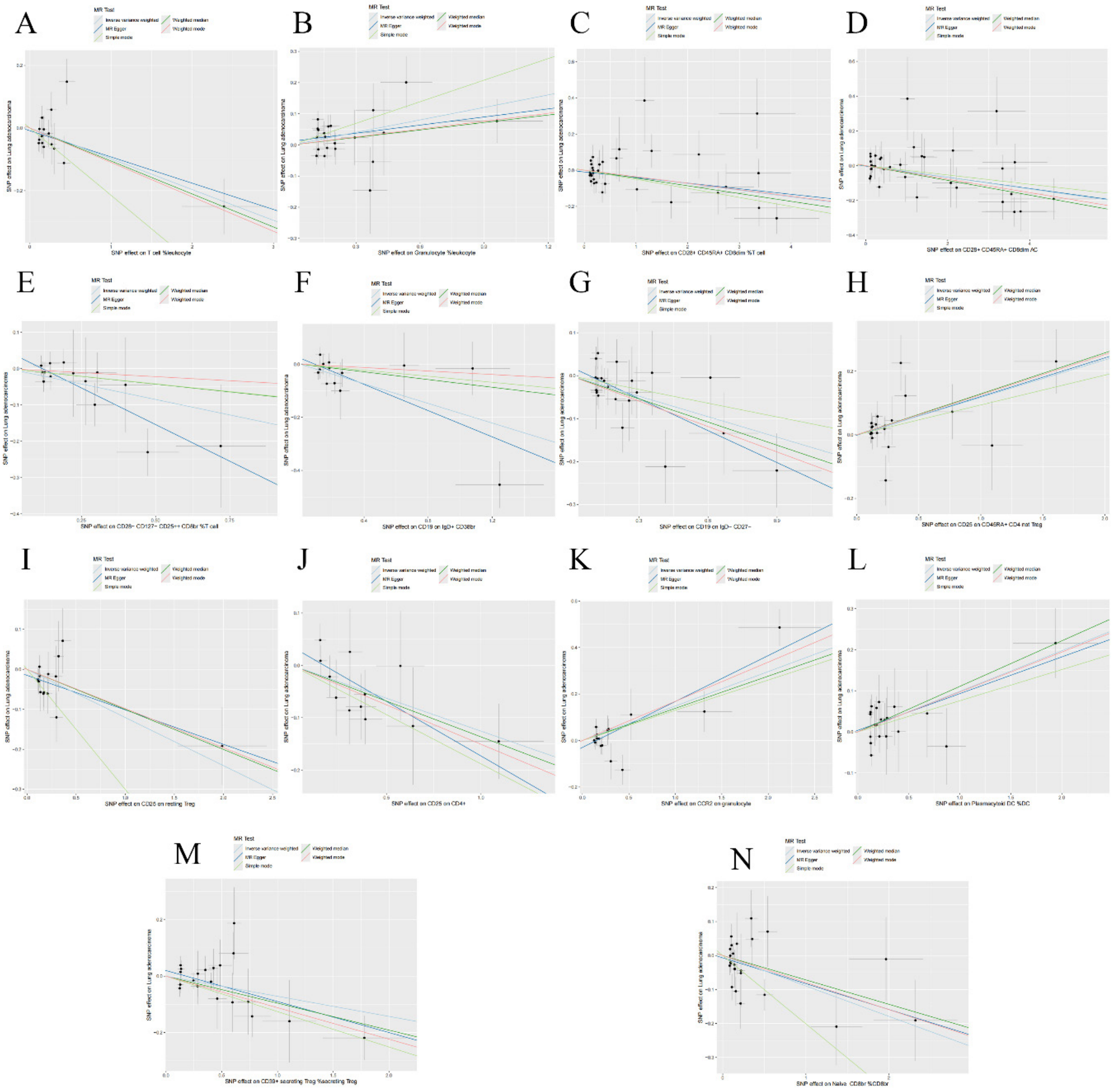

Figure S2 Leave-one-out plots for the causal association between immune cell traits and lung adenocarcinoma. Leave-one-out plots of Plasmacytoid DC %DC (A), CD39+ secreting Treg %secreting Treg (B), Naive CD8br %CD8br (C), T cell %leukocyte (D), Granulocyte %leukocyte (E), CD28+ CD45RA+ CD8dim %T cell (F), CD28+ CD45RA+ CD8dim AC (G), CD28- CD127- CD25++ CD8br %T cell (H), CD19 on IgD+ CD38br (I), CD19 on IgD- CD27- (J), CD25 on CD45RA+ CD4 not Treg (K), CD25 on resting Treg (L), CD25 on CD4+ (M) and CCR2 on granulocyte (N).

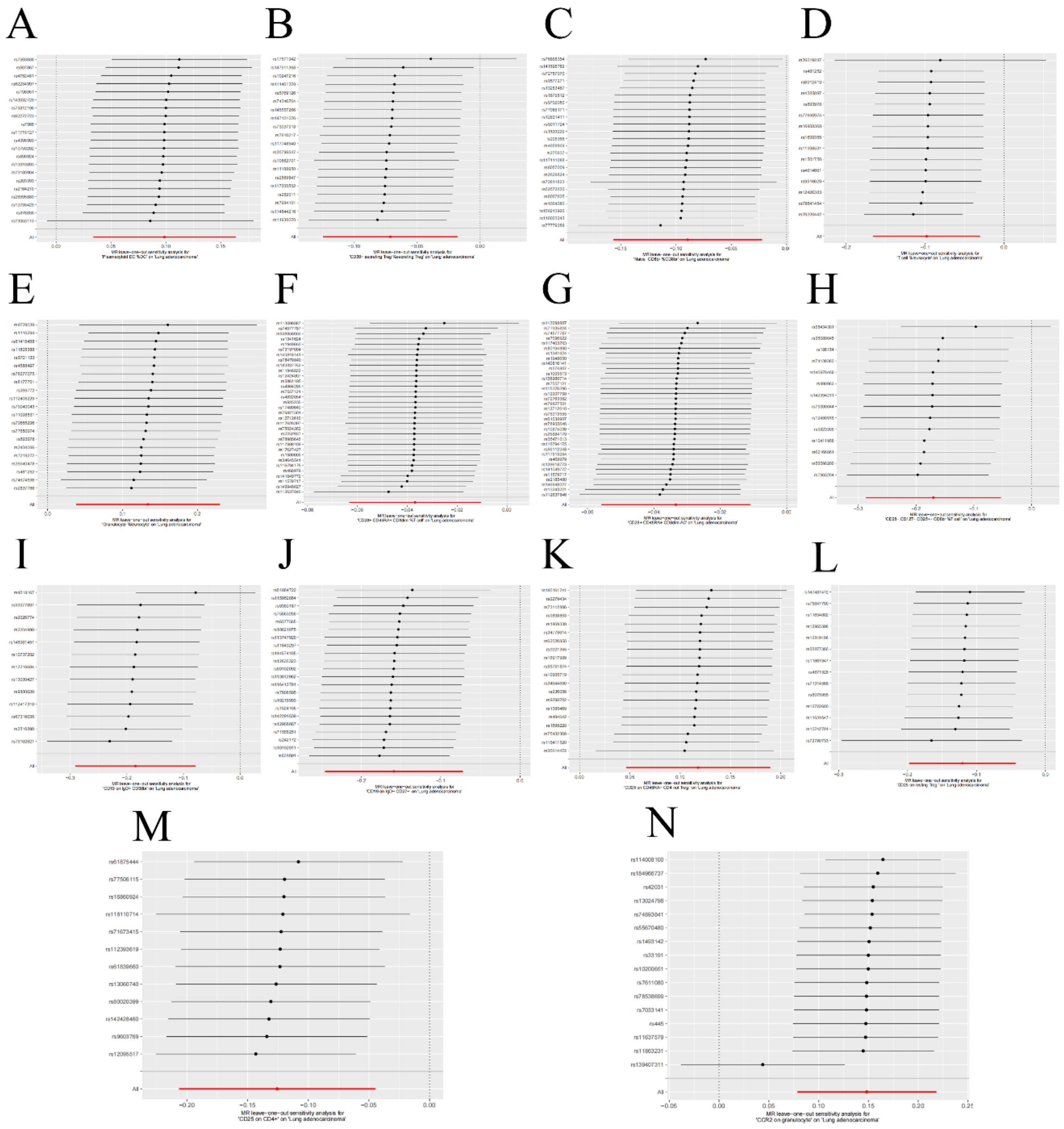

Figure S3 Dynamic circular heatmap of plasma metabolites.

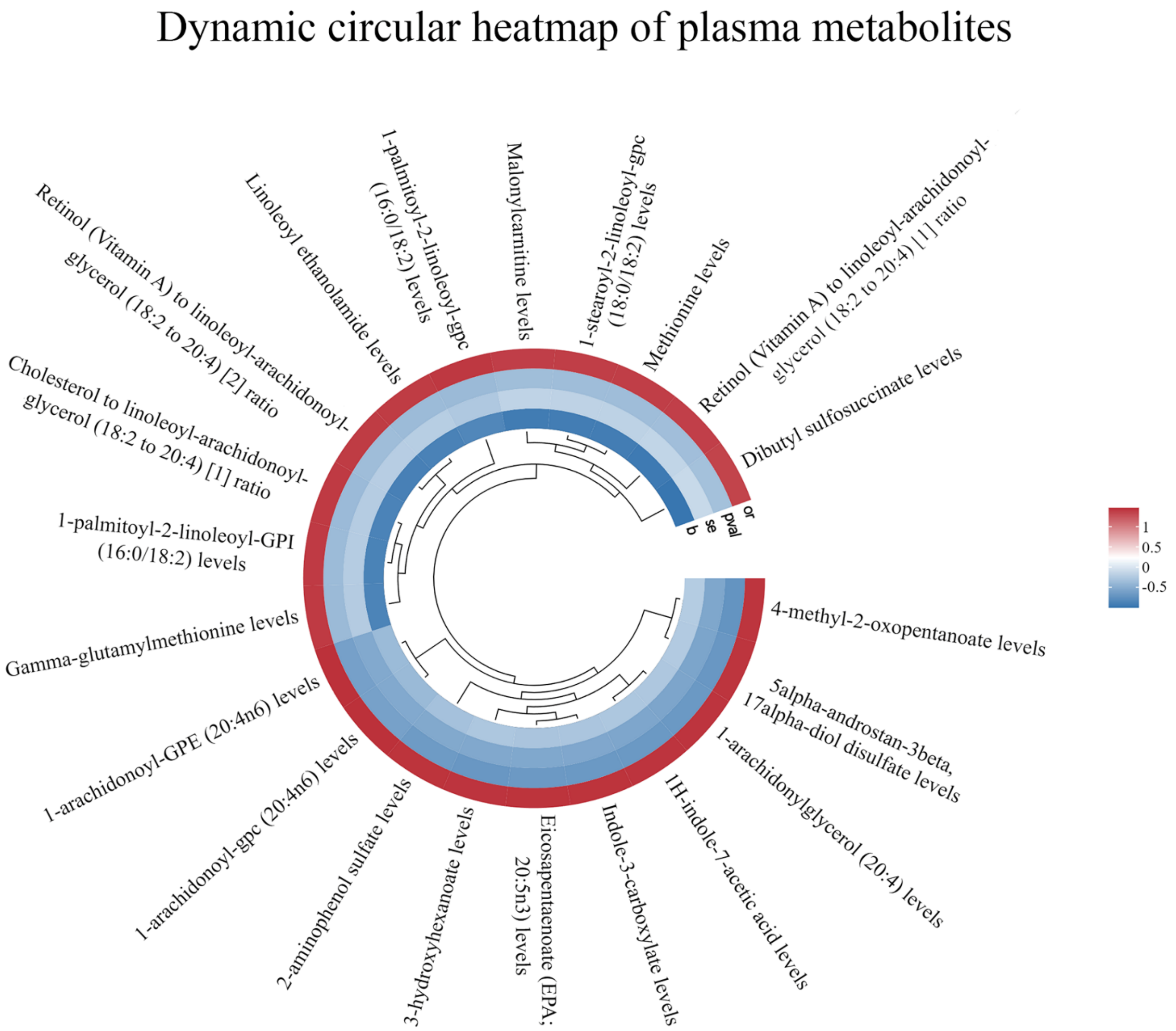

Figure S4 Scatter plots for the causal association between plasma metabolites and lung adenocarcinoma. Scatter plots of 4-methyl-2-oxopentanoate levels (A), Retinol (Vitamin A) to linoleoyl-arachidonoyl-glycerol (18:2 to 20:4) [1] ratio (B), Retinol (Vitamin A) to linoleoyl-arachidonoyl-glycerol (18:2 to 20:4) [2] ratio (C), Gamma-glutamylmethionine levels (D), 1-arachidonylglycerol (20:4) levels (E), Malonylcarnitine levels (F), 1-arachidonoyl-gpc (20:4n6) levels (G), 1-arachidonoyl-GPE (20:4n6) levels (H), Cholesterol to linoleoyl-arachidonoyl-glycerol (18:2 to 20:4) ratio (I), 5alpha-androstan-3beta,17alpha-diol disulfate levels (J), Indole-3-carboxylate levels (K), 2-aminophenol sulfate levels (L), 1H-indole-7-acetic acid levels (M), 3-hydroxyhexanoate levels (N), Linoleoyl ethanolamide levels (O), 1-stearoyl-2-linoleoyl-gpc (18:0/18:2) levels (P), Dibutyl sulfosuccinate levels (Q), 1-palmitoyl-2-linoleoyl-gpc (16:0/18:2) levels(R), 1-palmitoyl-2-linoleoyl-GPI (16:0/18:2) levels (S), Eicosapentaenoate (EPA; 20:5n3) levels (T) and Methionine levels (U).

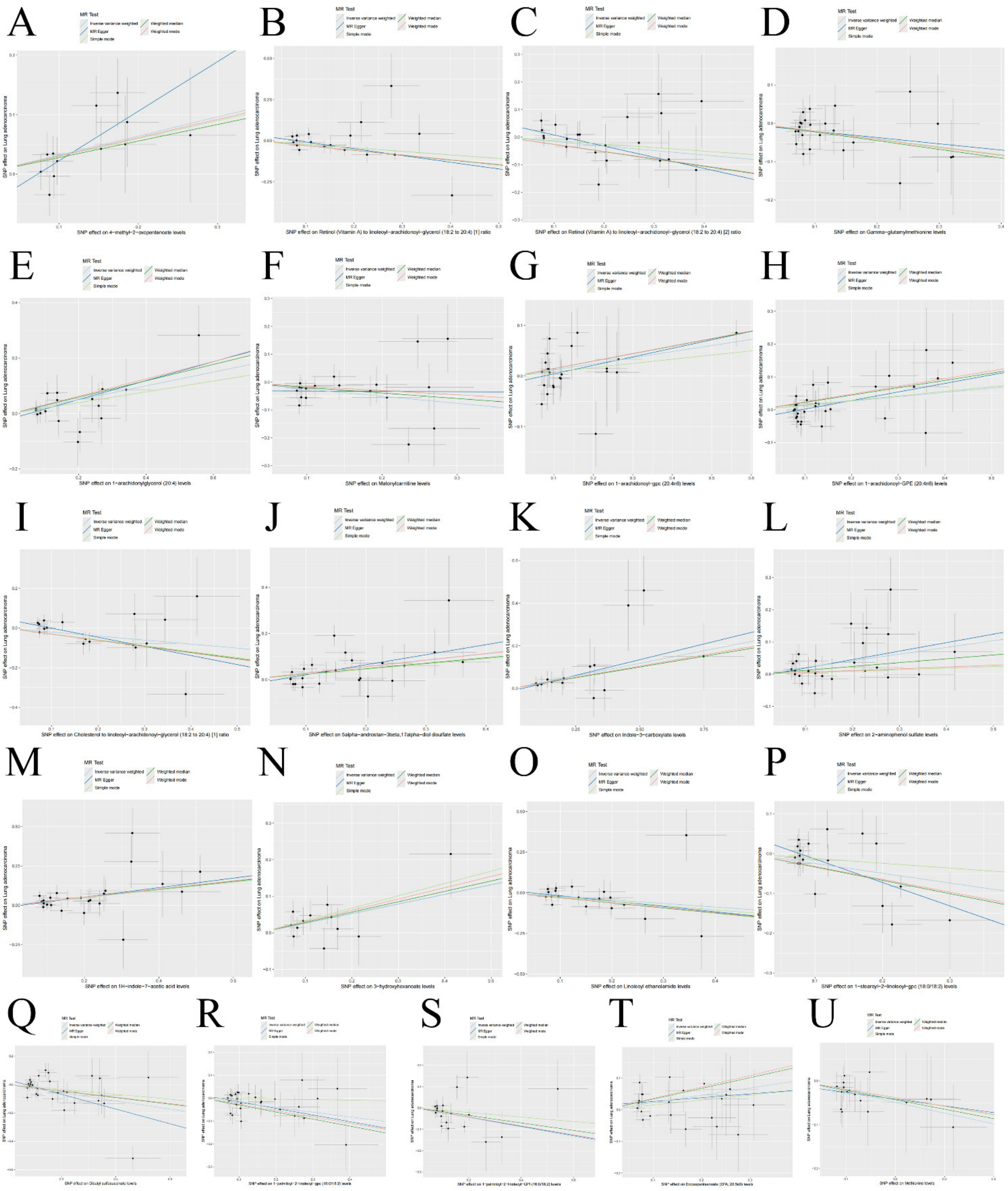
A
B
C
D
E
F
G
H
I
J
K
L
M
N
O
P
Q
R
S
T
U
MR Test
Inverse variance weighted
MR Egger
Simple mode
Weighted median
Weighted mode
SNP effect on Lung adenocarcinoma
SNP effect on 4-methyl-2-oxopentanoate levels
SNP effect on Retinol (Vitamin A) to linoleoyl-arachidonoyl-glycerol (18:2 to 20:4) [1] ratio
SNP effect on Retinol (Vitamin A) to linoleoyl-arachidonoyl-glycerol (18:2 to 20:4) [2] ratio
SNP effect on Gamma-glutamylmethionine levels
SNP effect on 1-arachidonylglycerol (20:4) levels
SNP effect on Malonylcarnitine levels
SNP effect on 1-arachidonoyl-gpc (20:4n6) levels
SNP effect on 1-arachidonoyl-GPE (20:4n6) levels
SNP effect on Cholesterol to linoleoyl-arachidonoyl-glycerol (18:2 to 20:4) [1] ratio
SNP effect on 5alpha-androstan-3beta,17alpha-diol disulfate levels
SNP effect on Indole-3-carboxylate levels
SNP effect on 2-aminophenol sulfate levels
SNP effect on 1H-indole-7-acetic acid levels
SNP effect on 3-hydroxyhexanoate levels
SNP effect on Linoleoyl ethanolamide levels
SNP effect on 1-stearoyl-2-linoleoyl-gpc (18:0/18:2) levels

Figure S5 Leave-one-out plots for the causal association between plasma metabolites and lung adenocarcinoma. Leave-one-out plots of 4-methyl-2-oxopentanoate levels (A), Retinol (Vitamin A) to linoleoyl-arachidonoyl-glycerol (18:2 to 20:4) [1] ratio (B), Retinol (Vitamin A) to linoleoyl-arachidonoyl-glycerol (18:2 to 20:4) [2] ratio (C), Gamma-glutamylmethionine levels (D), 1-arachidonylglycerol (20:4) levels (E), Malonylcarnitine levels (F), 1-arachidonoyl-gpc (20:4n6) levels (G), 1-arachidonoyl-GPE (20:4n6) levels (H), Cholesterol to linoleoyl-arachidonoyl-glycerol (18:2 to 20:4) ratio (I), 5alpha-androstan-3beta,17alpha-diol disulfate levels (J), Indole-3-carboxylate levels (K), 2-aminophenol sulfate levels (L), 1H-indole-7-acetic acid levels (M), 3-hydroxyhexanoate levels (N), Linoleoyl ethanolamide levels (O), 1-stearoyl-2-linoleoyl-gpc (18:0/18:2) levels (P), Dibutyl sulfosuccinate levels (Q), 1-palmitoyl-2-linoleoyl-gpc (16:0/18:2) levels(R), 1-palmitoyl-2-linoleoyl-GPI (16:0/18:2) levels (S), Eicosapentaenoate (EPA; 20:5n3) levels (T) and Methionine levels (U).

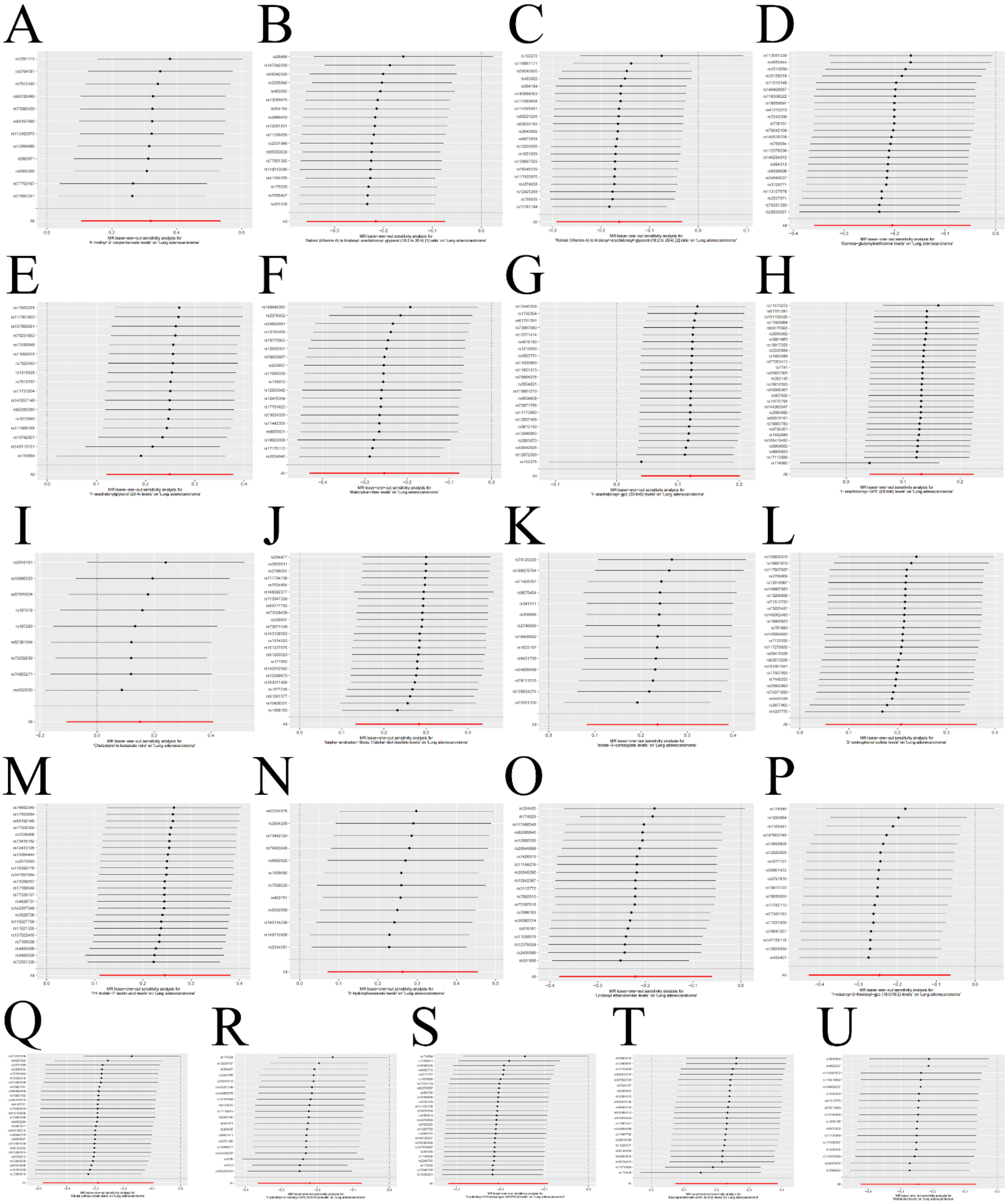
A
B
C
D
E
F
G
H
I
J
K
L
M
N
O
P
Q
R
S
T
U

Figure S6 Enrichment analysis results of the causal plasma metabolites of lung adenocarcinoma based on the Small Molecule Pathway Database. Bar chart (A), Dot Plot (B).

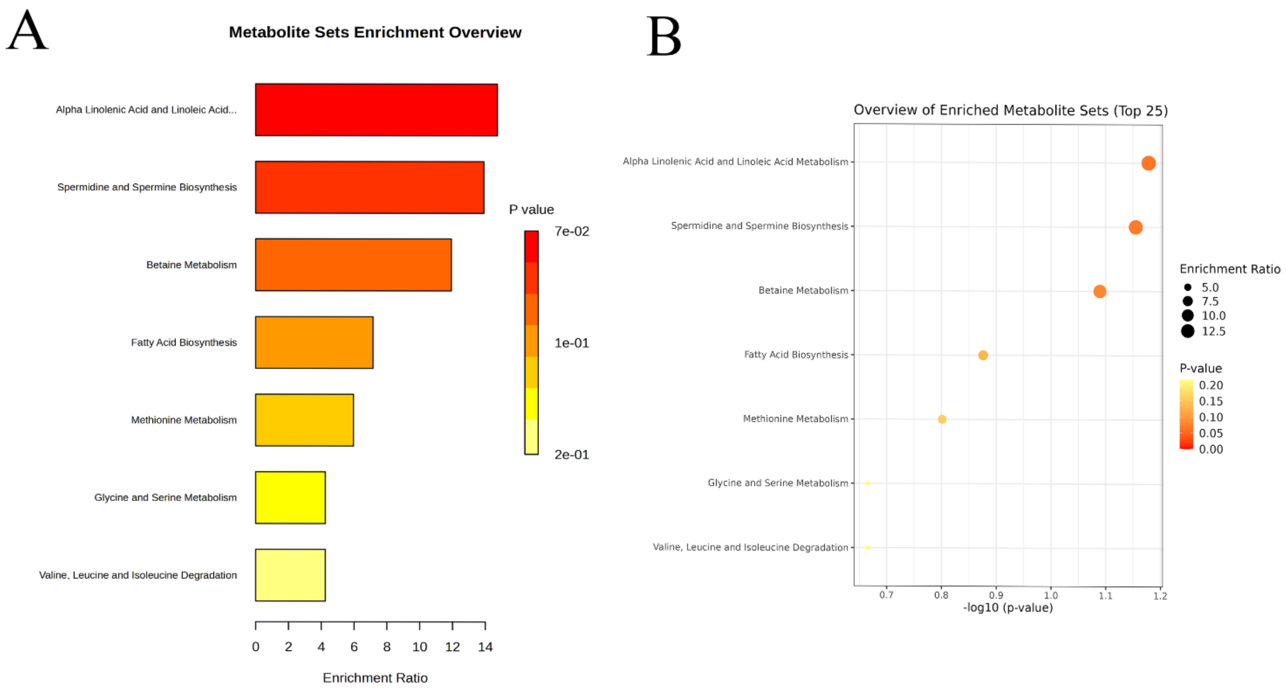

Figure S7 Forest plots of 9 mediation pathways. Forest plots of CD28- CD127- CD25++ CD8br %T cell via Methionine levels (A), CD28- CD127- CD25++ CD8br %T cell via 3-hydroxyhexanoate levels (B), T cell %leukocyte via Linoleoyl ethanolamide levels (C), CD25 on CD4+ via Methionine levels (D), CCR2 on granulocyte on LUAD via 5alpha-androstan-3beta, 17alpha-diol disulfate levels (E), CD28+ CD45RA+ CD8dim %T cell via Indole-3-carboxylate levels (F), CD28+ CD45RA+ CD8dim AC via Indole-3-carboxylate levels (G), CD39+ secreting Treg %secreting Treg via 1-arachidonylglycerol (20:4) levels (H) and CD39+ secreting Treg %secreting Treg via 1-stearoyl-2-linoleoyl-GPC (18:0/18:2) levels (I).

A

exposure	outcome	nsnp	method	pval	OR(95% CI)
CD28− CD127− CD25++ CD8br %T cell	GCST90200391	17	MR Egger	0.665	0.984 (0.916 to 1.057)
		17	Weighted median	0.119	0.947 (0.883 to 1.014)
		17	Inverse variance weighted	**0.023**	0.943 (0.897 to 0.992)
		17	Simple mode	**0.037**	0.861 (0.756 to 0.980)
		17	Weighted mode	0.093	0.941 (0.880 to 1.006)
Methionine levels	Lung adenocarcinoma	17	MR Egger	0.582	0.858 (0.503 to 1.463)
		17	Weighted median	0.076	0.806 (0.636 to 1.022)
		17	Inverse variance weighted	**0.007**	0.783 (0.654 to 0.937)
		17	Simple mode	0.358	0.824 (0.552 to 1.231)
		17	Weighted mode	0.327	0.826 (0.570 to 1.196)
CD28− CD127− CD25++ CD8br %T cell	Lung adenocarcinoma	14	MR Egger	**0.009**	0.668 (0.519 to 0.861)
		14	Weighted median	0.340	0.918 (0.770 to 1.094)
		14	Inverse variance weighted	**0.004**	0.843 (0.751 to 0.948)
		14	Simple mode	0.576	0.919 (0.689 to 1.226)
		14	Weighted mode	0.778	0.956 (0.706 to 1.296)

B

exposure	outcome	nsnp	method	pval	OR(95% CI)
CD28− CD127− CD25++ CD8br %T cell	GCST90200012	17	MR Egger	0.061	1.072 (1.002 to 1.147)
		17	Weighted median	0.133	1.053 (0.984 to 1.126)
		17	Inverse variance weighted	**0.039**	1.049 (1.002 to 1.097)
		17	Simple mode	0.445	1.046 (0.934 to 1.171)
		17	Weighted mode	0.149	1.054 (0.985 to 1.128)
3−hydroxyhexanoate levels	Lung adenocarcinoma	12	MR Egger	0.293	1.301 (0.817 to 2.071)
		12	Weighted median	**0.038**	1.328 (1.016 to 1.735)
		12	Inverse variance weighted	**0.007**	1.300 (1.074 to 1.574)
		12	Simple mode	0.124	1.401 (0.942 to 2.083)
		12	Weighted mode	0.129	1.361 (0.942 to 1.966)
CD28− CD127− CD25++ CD8br %T cell	Lung adenocarcinoma	14	MR Egger	**0.009**	0.668 (0.519 to 0.861)
		14	Weighted median	0.330	0.918 (0.773 to 1.090)
		14	Inverse variance weighted	**0.004**	0.843 (0.751 to 0.948)
		14	Simple mode	0.585	0.919 (0.684 to 1.235)
		14	Weighted mode	0.798	0.956 (0.685 to 1.335)

C

exposure	outcome	nsnp	method	pval	OR(95% CI)
T cell %leukocyte	GCST90200029	17	MR Egger	**0.035**	1.063 (1.010 to 1.120)
		17	Weighted median	0.050	1.061 (1.000 to 1.125)
		17	Inverse variance weighted	**0.014**	1.053 (1.011 to 1.096)
		17	Simple mode	0.069	1.141 (0.999 to 1.304)
		17	Weighted mode	**0.030**	1.068 (1.012 to 1.127)
Linoleoyl ethanolamide levels	Lung adenocarcinoma	20	MR Egger	0.109	0.720 (0.491 to 1.055)
		20	Weighted median	**0.005**	0.731 (0.588 to 0.910)
		20	Inverse variance weighted	**0.007**	0.802 (0.683 to 0.941)
		20	Simple mode	0.171	0.768 (0.534 to 1.105)
		20	Weighted mode	**0.022**	0.736 (0.579 to 0.937)
T cell %leukocyte	Lung adenocarcinoma	15	MR Egger	0.074	0.920 (0.845 to 1.001)
		15	Weighted median	**0.028**	0.900 (0.820 to 0.988)
		15	Inverse variance weighted	**0.004**	0.906 (0.847 to 0.970)
		15	Simple mode	0.080	0.807 (0.645 to 1.008)
		15	Weighted mode	**0.020**	0.896 (0.825 to 0.973)

D

exposure	outcome	nsnp	method	pval	OR(95% CI)
CD25 on CD4+	GCST90200391	15	MR Egger	0.407	1.017 (0.978 to 1.059)
		15	Weighted median	0.213	1.026 (0.985 to 1.069)
		15	Inverse variance weighted	**0.028**	1.035 (1.004 to 1.068)
		15	Simple mode	0.160	1.053 (0.984 to 1.128)
		15	Weighted mode	0.188	1.027 (0.989 to 1.066)
Methionine levels	Lung adenocarcinoma	17	MR Egger	0.582	0.858 (0.503 to 1.463)
		17	Weighted median	0.075	0.806 (0.636 to 1.022)
		17	Inverse variance weighted	**0.007**	0.783 (0.654 to 0.937)
		17	Simple mode	0.344	0.824 (0.558 to 1.216)
		17	Weighted mode	0.383	0.826 (0.544 to 1.254)
CD25 on CD4+	Lung adenocarcinoma	12	MR Egger	**0.014**	0.812 (0.707 to 0.932)
		12	Weighted median	**0.025**	0.872 (0.774 to 0.983)
		12	Inverse variance weighted	**0.002**	0.882 (0.813 to 0.956)
		12	Simple mode	**0.048**	0.828 (0.702 to 0.978)
		12	Weighted mode	**0.039**	0.860 (0.758 to 0.975)

E

exposure	outcome	nsnp	method	pval	OR(95% CI)
CCR2 on granulocyte	GCST90199852	17	MR Egger	0.123	1.044 (0.991 to 1.100)
		17	Weighted median	0.227	1.035 (0.979 to 1.095)
		17	Inverse variance weighted	**0.036**	1.042 (1.003 to 1.082)
		17	Simple mode	0.110	1.073 (0.989 to 1.165)
		17	Weighted mode	0.165	1.040 (0.987 to 1.095)
5alpha−androstan−3beta,17alpha−diol disulfate levels	Lung adenocarcinoma	23	MR Egger	**0.024**	1.546 (1.088 to 2.198)
		23	Weighted median	**0.018**	1.267 (1.042 to 1.541)
		23	Inverse variance weighted	**<0.001**	1.327 (1.142 to 1.542)
		23	Simple mode	0.160	1.256 (0.924 to 1.707)
		23	Weighted mode	**0.048**	1.324 (1.018 to 1.722)
CCR2 on granulocyte	Lung adenocarcinoma	16	MR Egger	**<0.001**	1.218 (1.123 to 1.322)
		16	Weighted median	**0.011**	1.149 (1.032 to 1.278)
		16	Inverse variance weighted	**<0.001**	1.160 (1.082 to 1.243)
		16	Simple mode	0.244	1.139 (0.923 to 1.405)
		16	Weighted mode	**0.003**	1.184 (1.078 to 1.300)

F

exposure	outcome	nsnp	method	pval	OR(95% CI)
CD28+ CD45RA+ CD8dim %T cell	GCST90199877	37	MR Egger	0.168	1.012 (0.995 to 1.029)
		37	Weighted median	0.432	1.008 (0.988 to 1.028)
		37	Inverse variance weighted	**0.046**	1.015 (1.000 to 1.030)
		37	Simple mode	0.859	1.003 (0.975 to 1.031)
		37	Weighted mode	0.281	1.010 (0.992 to 1.029)
Indole−3−carboxylate levels	Lung adenocarcinoma	14	MR Egger	0.075	1.343 (0.999 to 1.805)
		14	Weighted median	**0.047**	1.223 (1.003 to 1.490)
		14	Inverse variance weighted	**0.003**	1.266 (1.086 to 1.476)
		14	Simple mode	0.195	1.233 (0.913 to 1.665)
		14	Weighted mode	0.197	1.233 (0.912 to 1.667)
CD28+ CD45RA+ CD8dim %T cell	Lung adenocarcinoma	33	MR Egger	**0.048**	0.970 (0.941 to 0.999)
		33	Weighted median	**0.016**	0.958 (0.925 to 0.992)
		33	Inverse variance weighted	**0.006**	0.964 (0.939 to 0.990)
		33	Simple mode	0.146	0.951 (0.890 to 1.016)
		33	Weighted mode	**0.010**	0.965 (0.940 to 0.990)

G

exposure	outcome	nsnp	method	pval	OR(95% CI)
CD28+ CD45RA+ CD8dim AC	GCST90199877	47	MR Egger	**0.038**	1.012 (1.001 to 1.023)
		47	Weighted median	0.501	1.005 (0.991 to 1.019)
		47	Inverse variance weighted	**0.003**	1.014 (1.005 to 1.024)
		47	Simple mode	0.626	1.004 (0.987 to 1.022)
		47	Weighted mode	0.368	1.006 (0.993 to 1.019)
Indole−3−carboxylate levels	Lung adenocarcinoma	14	MR Egger	0.075	1.343 (0.999 to 1.805)
		14	Weighted median	0.058	1.223 (0.993 to 1.505)
		14	Inverse variance weighted	**0.003**	1.266 (1.086 to 1.476)
		14	Simple mode	0.211	1.233 (0.903 to 1.684)
		14	Weighted mode	0.172	1.233 (0.928 to 1.637)
CD28+ CD45RA+ CD8dim AC	Lung adenocarcinoma	35	MR Egger	**0.019**	0.968 (0.944 to 0.993)
		35	Weighted median	**0.003**	0.959 (0.932 to 0.985)
		35	Inverse variance weighted	**0.003**	0.967 (0.946 to 0.989)
		35	Simple mode	0.208	0.974 (0.935 to 1.014)
		35	Weighted mode	**<0.001**	0.962 (0.942 to 0.982)

H

exposure	outcome	nsnp	method	pval	OR(95% CI)
CD39+ secreting Treg %secreting Treg	GCST90199770	27	MR Egger	0.120	0.970 (0.935 to 1.007)
		27	Weighted median	0.103	0.967 (0.929 to 1.007)
		27	Inverse variance weighted	**0.036**	0.973 (0.948 to 0.998)
		27	Simple mode	0.503	0.979 (0.922 to 1.040)
		27	Weighted mode	0.060	0.958 (0.917 to 1.000)
1−arachidonylglycerol (20:4) levels	Lung adenocarcinoma	17	MR Egger	**0.013**	1.407 (1.110 to 1.784)
		17	Weighted median	**<0.001**	1.343 (1.129 to 1.598)
		17	Inverse variance weighted	**<0.001**	1.283 (1.128 to 1.459)
		17	Simple mode	0.260	1.215 (0.876 to 1.687)
		17	Weighted mode	**0.004**	1.366 (1.140 to 1.638)
CD39+ secreting Treg %secreting Treg	Lung adenocarcinoma	20	MR Egger	**0.012**	0.896 (0.830 to 0.967)
		20	Weighted median	**0.011**	0.909 (0.844 to 0.978)
		20	Inverse variance weighted	**0.009**	0.931 (0.883 to 0.982)
		20	Simple mode	0.095	0.882 (0.767 to 1.015)
		20	Weighted mode	**0.013**	0.894 (0.825 to 0.969)

I

exposure	outcome	nsnp	method	pval	OR(95% CI)
CD39+ secreting Treg %secreting Treg	GCST90200037	27	MR Egger	**0.038**	0.962 (0.929 to 0.996)
		27	Weighted median	0.147	0.974 (0.940 to 1.009)
		27	Inverse variance weighted	**0.042**	0.974 (0.950 to 0.999)
		27	Simple mode	0.131	0.958 (0.908 to 1.011)
		27	Weighted mode	0.203	0.976 (0.941 to 1.012)
1−stearoyl−2−linoleoyl−gpc (18:0/18:2) levels	Lung adenocarcinoma	18	MR Egger	**0.007**	0.554 (0.380 to 0.808)
		18	Weighted median	**0.001**	0.715 (0.582 to 0.879)
		18	Inverse variance weighted	**0.008**	0.782 (0.652 to 0.938)
		18	Simple mode	0.663	0.886 (0.519 to 1.512)
		18	Weighted mode	**0.014**	0.724 (0.576 to 0.912)
CD39+ secreting Treg %secreting Treg	Lung adenocarcinoma	20	MR Egger	**0.012**	0.896 (0.830 to 0.967)
		20	Weighted median	**0.020**	0.909 (0.838 to 0.985)
		20	Inverse variance weighted	**0.009**	0.931 (0.883 to 0.982)
		20	Simple mode	0.114	0.882 (0.760 to 1.023)
		20	Weighted mode	**0.019**	0.894 (0.821 to 0.974)

Table S1 The heterogeneity analysis of causality between immune cells and lung adenocarcinoma based on IVW and MR Egger methods.

Exposure	Method	Q	Q_df	Q_pval
Plasmacytoid DC %DC	MR Egger	22.59	19.00	0.26
Plasmacytoid DC %DC	Inverse variance weighted	22.69	20.00	0.30
CD39+ secreting Treg %secreting Treg	MR Egger	16.58	18.00	0.55
CD39+ secreting Treg %secreting Treg	Inverse variance weighted	18.44	19.00	0.49
Naive CD8br %CD8br	MR Egger	30.38	22.00	0.11
Naive CD8br %CD8br	Inverse variance weighted	30.83	23.00	0.13
T cell %leukocyte	MR Egger	16.60	13.00	0.22
T cell %leukocyte	Inverse variance weighted	17.03	14.00	0.25
Granulocyte %leukocyte	MR Egger	26.76	19.00	0.11
Granulocyte %leukocyte	Inverse variance weighted	27.37	20.00	0.13
CD28+ CD45RA+ CD8dim %T cell	MR Egger	42.83	31.00	0.08
CD28+ CD45RA+ CD8dim %T cell	Inverse variance weighted	43.92	32.00	0.08
CD28+ CD45RA+ CD8dim AC	MR Egger	56.99	33.00	5.88E-03
CD28+ CD45RA+ CD8dim AC	Inverse variance weighted	57.05	34.00	7.93E-03
CD28- CD127- CD25++ CD8br %T cell	MR Egger	7.74	12.00	0.80
CD28- CD127- CD25++ CD8br %T cell	Inverse variance weighted	11.87	13.00	0.54
CD19 on IgD+ CD38br	MR Egger	17.50	11.00	0.09
CD19 on IgD+ CD38br	Inverse variance weighted	19.43	12.00	0.08
CD19 on IgD- CD27-	MR Egger	18.41	20.00	0.56
CD19 on IgD- CD27-	Inverse variance weighted	20.31	21.00	0.50
CD25 on CD45RA+ CD4 not Treg	MR Egger	16.71	18.00	0.54
CD25 on CD45RA+ CD4 not Treg	Inverse variance weighted	16.71	19.00	0.61
CD25 on resting Treg	MR Egger	8.19	12.00	0.77
CD25 on resting Treg	Inverse variance weighted	9.26	13.00	0.75
CD25 on CD4+	MR Egger	6.76	10.00	0.75
CD25 on CD4+	Inverse variance weighted	8.87	11.00	0.63
CCR2 on granulocyte	MR Egger	19.61	14.00	0.14
CCR2 on granulocyte	Inverse variance weighted	24.68	15.00	0.05

Abbreviations: IVW: Inverse-variance weighted; LUAD: Lung adenocarcinoma; MR: Mendelian randomization.

Table S2 The pleiotropy analysis of causality between immune cells and lung adenocarcinoma based on MR results.

Exposure	Egger intercept	Se	P-value
CD86+ plasmacytoid DC %DC	0.01	0.02	0.80
CD39+ secreting Treg %secreting Treg	0.02	0.01	0.19
Naive CD8br %CD8br	-0.01	0.01	0.58
T cell %leukocyte	-0.01	0.01	0.57
Granulocyte %leukocyte	0.01	0.02	0.52
CD28+ CD45RA+ CD8dim %T cell	-0.01	0.01	0.38
CD28+ CD45RA+ CD8dim AC	0.00	0.01	0.85
CD28- CD127- CD25++ CD8br %T cell	0.05	0.02	0.07
CD19 on IgD+ CD38br	0.02	0.02	0.29
CD19 on IgD- CD27-	0.02	0.02	0.18
CD25 on CD45RA+ CD4 not Treg	0.00	0.01	0.93
CD25 on resting Treg	-0.01	0.01	0.32
CD25 on CD4+	0.04	0.02	0.18
CCR2 on granulocyte	-0.03	0.02	0.08

Abbreviations: LUAD: Lung adenocarcinoma; MR: Mendelian randomization; Se: Standard error.

Table S3 Reverse Mendelian randomization analysis of lung adenocarcinoma on immune cells.

Exposure	Method	Nsnp	B	Se	OR	Rev P-value
Plasmacytoid DC %DC	IVW	21	0.10	0.03	1.10	0.73
CD39+ secreting Treg %secreting Treg	IVW	20	-0.07	0.03	0.93	0.61
Naive CD8br %CD8br	IVW	24	-0.09	0.03	0.91	0.65
T cell %leukocyte	IVW	15	-0.10	0.03	0.91	0.56
Granulocyte %leukocyte	IVW	21	0.13	0.05	1.14	0.39
CD28+ CD45RA+ CD8dim %T cell	IVW	33	-0.04	0.01	0.96	0.81
CD28+ CD45RA+ CD8dim AC	IVW	35	-0.03	0.01	0.97	0.59
CD28- CD127- CD25++ CD8br %T cell	IVW	14	-0.17	0.06	0.84	0.20
CD19 on IgD+ CD38br	IVW	13	-0.18	0.05	0.83	0.42
CD19 on IgD- CD27-	IVW	22	-0.16	0.04	0.85	0.76
CD25 on CD45RA+ CD4 not Treg	IVW	20	0.12	0.04	1.12	0.86
CD25 on resting Treg	IVW	14	-0.12	0.04	0.89	0.83
CD25 on CD4+	IVW	12	-0.13	0.04	0.88	0.58
CCR2 on granulocyte	IVW	16	0.15	0.04	1.16	0.48

Abbreviations: B: Beta-value; IVW: Inverse-variance weighted; LUAD: Lung adenocarcinoma; Nsnp: Number of SNPs; OR: Odds ratio; Snp: Single nucleotide polymorphism; Se: Standard error.

Table S4 The heterogeneity analysis of causality between plasma metabolites and lung adenocarcinoma based on IVW and MR Egger methods.

Exposure	Method	Q	Q_df	Q_pval
1-arachidonoyl-gpc (20:4n6) levels	Inverse variance weighted	23.09	22.00	0.40
1-arachidonoyl-gpc (20:4n6) levels	MR Egger	21.75	21.00	0.41
1-arachidonoyl-GPE (20:4n6) levels	Inverse variance weighted	25.85	28.00	0.58
1-arachidonoyl-GPE (20:4n6) levels	MR Egger	22.22	27.00	0.73
1-arachidonylglycerol (20:4) levels	Inverse variance weighted	13.25	16.00	0.65
1-arachidonylglycerol (20:4) levels	MR Egger	12.43	15.00	0.65
1H-indole-7-acetic acid levels	Inverse variance weighted	19.81	23.00	0.65
1H-indole-7-acetic acid levels	MR Egger	19.61	22.00	0.61
1-palmitoyl-2-linoleoyl-gpc (16:0/18:2) levels	Inverse variance weighted	24.69	26.00	0.54
1-palmitoyl-2-linoleoyl-gpc (16:0/18:2) levels	MR Egger	24.01	25.00	0.52
1-palmitoyl-2-linoleoyl-GPI (16:0/18:2) levels	Inverse variance weighted	24.00	19.00	0.20
1-palmitoyl-2-linoleoyl-GPI (16:0/18:2) levels	MR Egger	24.00	18.00	0.16
1-stearoyl-2-linoleoyl-gpc (18:0/18:2) levels	Inverse variance weighted	29.51	17.00	0.03
1-stearoyl-2-linoleoyl-gpc (18:0/18:2) levels	MR Egger	23.61	16.00	0.10
2-aminophenol sulfate levels	Inverse variance weighted	18.62	24.00	0.77
2-aminophenol sulfate levels	MR Egger	18.48	23.00	0.73
3-hydroxyhexanoate levels	Inverse variance weighted	7.54	11.00	0.75
3-hydroxyhexanoate levels	MR Egger	7.54	10.00	0.67
4-methyl-2-oxopentanoate levels	Inverse variance weighted	8.69	11.00	0.65
4-methyl-2-oxopentanoate levels	MR Egger	6.11	10.00	0.81
5alpha-androstan-3beta,17alpha-diol disulfate levels	Inverse variance weighted	30.38	22.00	0.11
5alpha-androstan-3beta,17alpha-diol disulfate levels	MR Egger	29.14	21.00	0.11
Cholesterol to linoleoyl-arachidonoyl-glycerol (18:2 to 20:4) [1] ratio	Inverse variance weighted	19.23	15.00	0.20
Cholesterol to linoleoyl-arachidonoyl-glycerol (18:2 to 20:4) [1] ratio	MR Egger	14.71	14.00	0.40
Dibutyl sulfosuccinate levels	Inverse variance weighted	63.29	27.00	9.61E-05
Dibutyl sulfosuccinate levels	MR Egger	59.07	26.00	2.23E-04
Eicosapentaenoate (EPA; 20:5n3) levels	Inverse variance weighted	25.32	21.00	0.23
Eicosapentaenoate (EPA; 20:5n3) levels	MR Egger	24.69	20.00	0.21
Gamma-glutamylmethionine levels	Inverse variance weighted	21.40	23.00	0.56
Gamma-glutamylmethionine levels	MR Egger	21.36	22.00	0.50
Indole-3-carboxylate levels	Inverse variance weighted	9.41	13.00	0.74
Indole-3-carboxylate levels	MR Egger	9.20	12.00	0.69
Linoleoyl ethanolamide levels	Inverse variance weighted	24.36	19.00	0.18
Linoleoyl ethanolamide levels	MR Egger	23.87	18.00	0.16

Malonylcarnitine levels	Inverse variance weighted	24.44	18.00	0.14
Malonylcarnitine levels	MR Egger	23.02	17.00	0.15
Methionine levels	Inverse variance weighted	6.75	16.00	0.98
Methionine levels	MR Egger	6.62	15.00	0.97
Retinol (Vitamin A) to linoleoyl-arachidonoyl-glycerol (18:2 to 20:4) [1] ratio	Inverse variance weighted	15.40	8.00	0.05
Retinol (Vitamin A) to linoleoyl-arachidonoyl-glycerol (18:2 to 20:4) [1] ratio	MR Egger	10.54	7.00	0.16
Retinol (Vitamin A) to linoleoyl-arachidonoyl-glycerol (18:2 to 20:4) [2] ratio	Inverse variance weighted	12.52	11.00	0.33
Retinol (Vitamin A) to linoleoyl-arachidonoyl-glycerol (18:2 to 20:4) [2] ratio	MR Egger	10.44	10.00	0.40

Abbreviations: IVW: inverse-variance weighted; MR: Mendelian randomization; LUAD: Lung adenocarcinoma.

Note: [1] The data comes from the GCST90200907 dataset;

[2] The data comes from the GCST90200908 dataset.

Table S5 The pleiotropy analysis of causality between plasma metabolites and lung adenocarcinoma based on MR results.

Exposure	Egger intercept	Se	P-value
4-methyl-2-oxopentanoate levels	-0.06	0.04	0.14
Gamma-glutamylmethionine levels	-4.43E-03	0.02	0.83
1-arachidonylglycerol (20:4) levels	-0.02	0.02	0.38
Malonylcarnitine levels	-0.03	0.03	0.32
1-arachidonoyl-gpc (20:4n6) levels	-0.01	0.01	0.27
1-arachidonoyl-GPE (20:4n6) levels	-0.02	0.01	0.07
5alpha-androstan-3beta,17alpha-diol disulfate levels	-0.02	0.02	0.36
Indole-3-carboxylate levels	-0.01	0.03	0.66
2-aminophenol sulfate levels	-7.44E-03	0.02	0.72
1H-indole-7-acetic acid levels	-8.86E-03	0.02	0.66
3-hydroxyhexanoate levels	-5.78E-05	0.03	1.00
Linoleoyl ethanolamide levels	0.01	0.02	0.55
1-stearoyl-2-linoleoyl-gpc (18:0/18:2) levels	0.05	0.02	0.06
Dibutyl sulfosuccinate levels	0.03	0.03	0.18
1-palmitoyl-2-linoleoyl-gpc (16:0/18:2) levels	0.01	0.02	0.42
1-palmitoyl-2-linoleoyl-GPI (16:0/18:2) levels	-1.10E-03	0.02	0.96
Eicosapentaenoate (EPA; 20:5n3) levels	0.01	0.02	0.48
Methionine levels	-0.01	0.03	0.73
Retinol (Vitamin A) to linoleoyl-arachidonoyl-glycerol (18:2 to 20:4) [1] ratio	0.07	0.04	0.12
Retinol (Vitamin A) to linoleoyl-arachidonoyl-glycerol (18:2 to 20:4) [2] ratio	0.03	0.02	0.19
Cholesterol to linoleoyl-arachidonoyl-glycerol (18:2 to 20:4) [1] ratio	0.04	0.02	0.06

Abbreviations: LUAD: Lung adenocarcinoma; MR: Mendelian randomization; Se: Standard error.

Note: [1] The data comes from the GCST90200907 dataset;

[2] The data comes from the GCST90200908 dataset.
