# Supplementary figures and images for: Causal relationships between immune cells, plasma metabolites and lung adenocarcinoma: a two-step, two-sample Mendelian randomization study
Source: J Cancer. 2024 Oct 28;15(20):6698–709. doi: 10.7150/jca.102760 (PMC11632987; doi:10.7150/jca.102760)

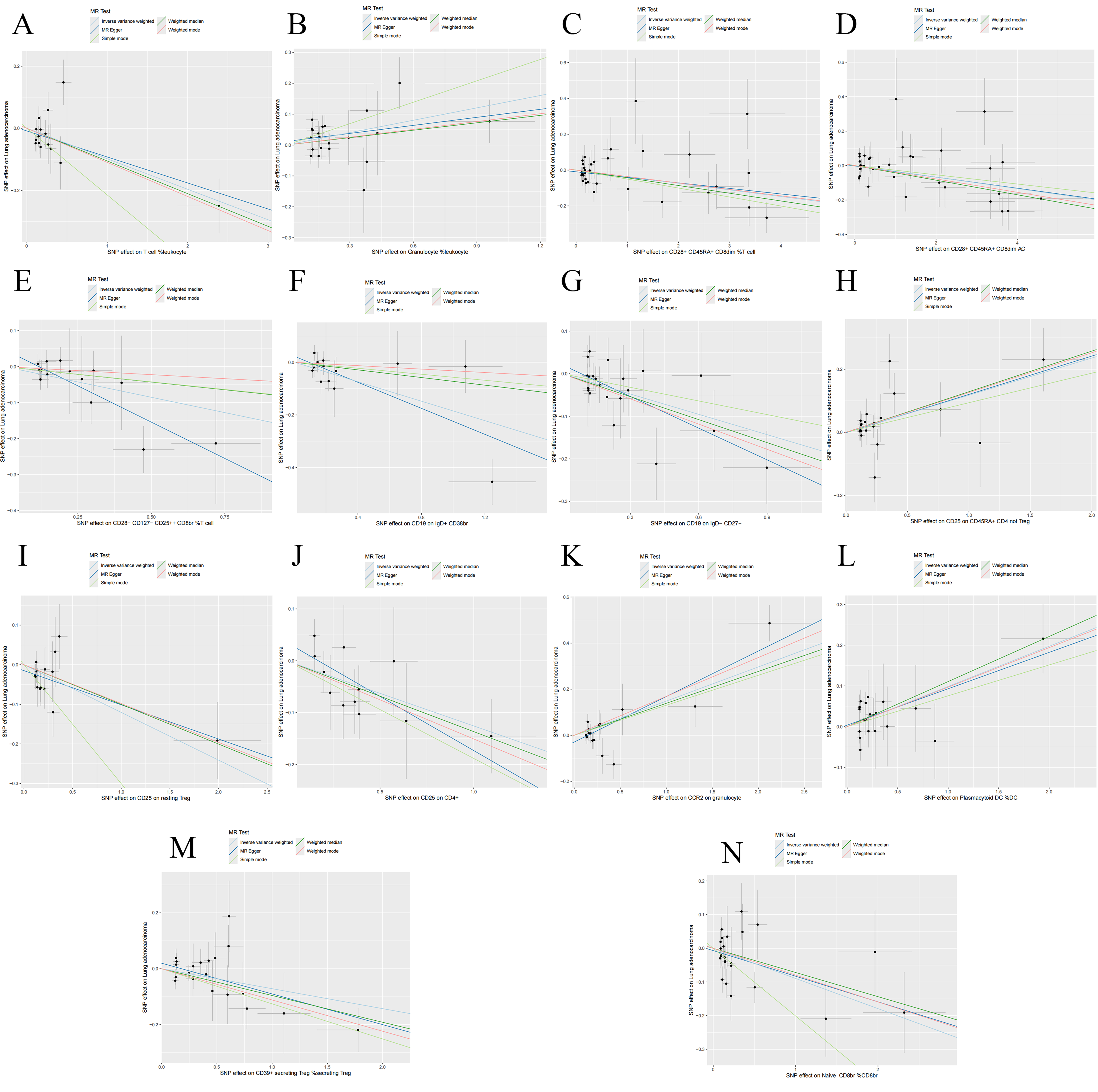

Supplement: Supplementary file 2 — Supplementary figures only (higher quality). [file jcav15p6698s2.zip › S-Figures/Figure S1.tif]

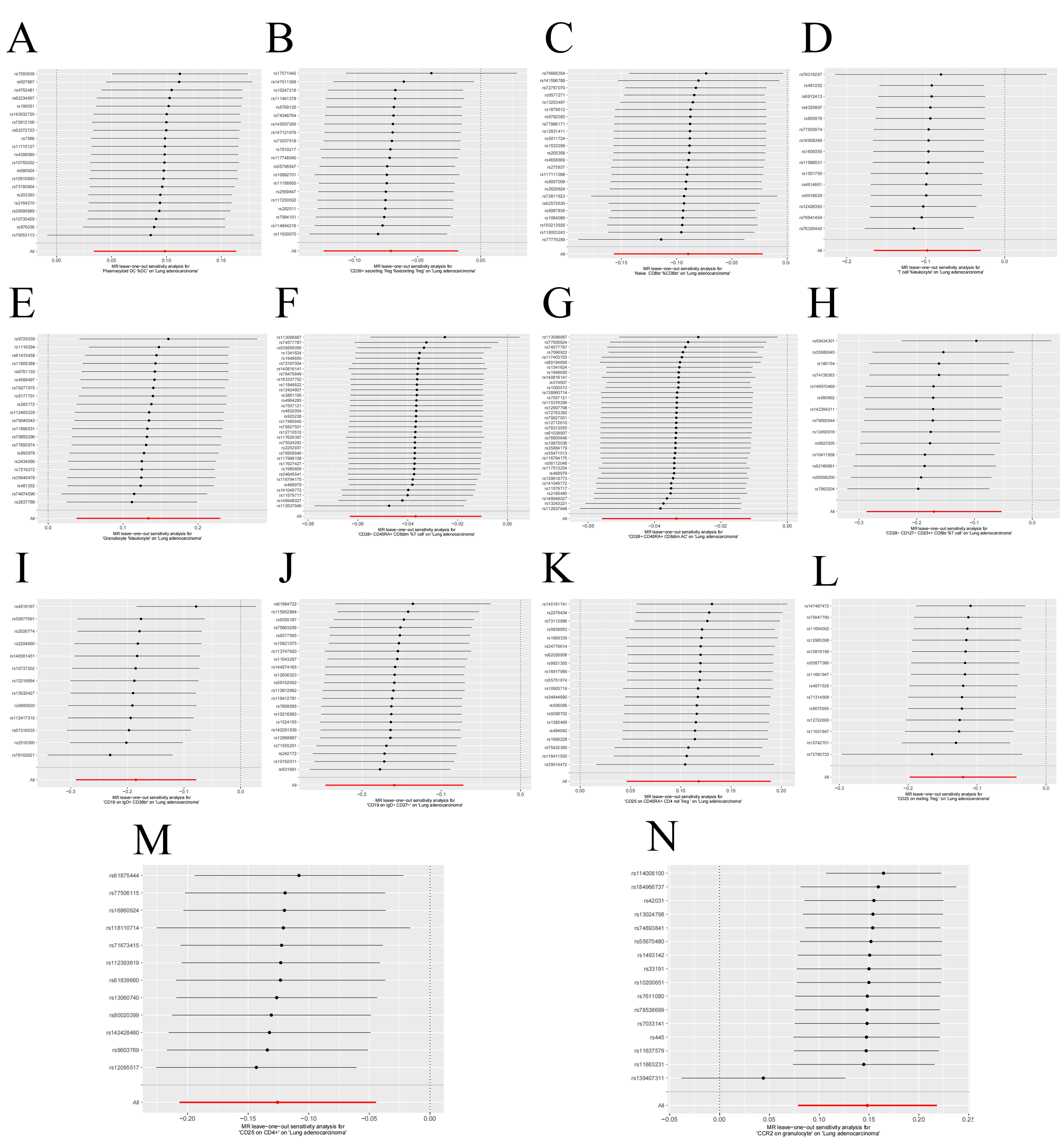

Supplement: Supplementary file 2 — Supplementary figures only (higher quality). [file jcav15p6698s2.zip › S-Figures/Figure S2.tif]

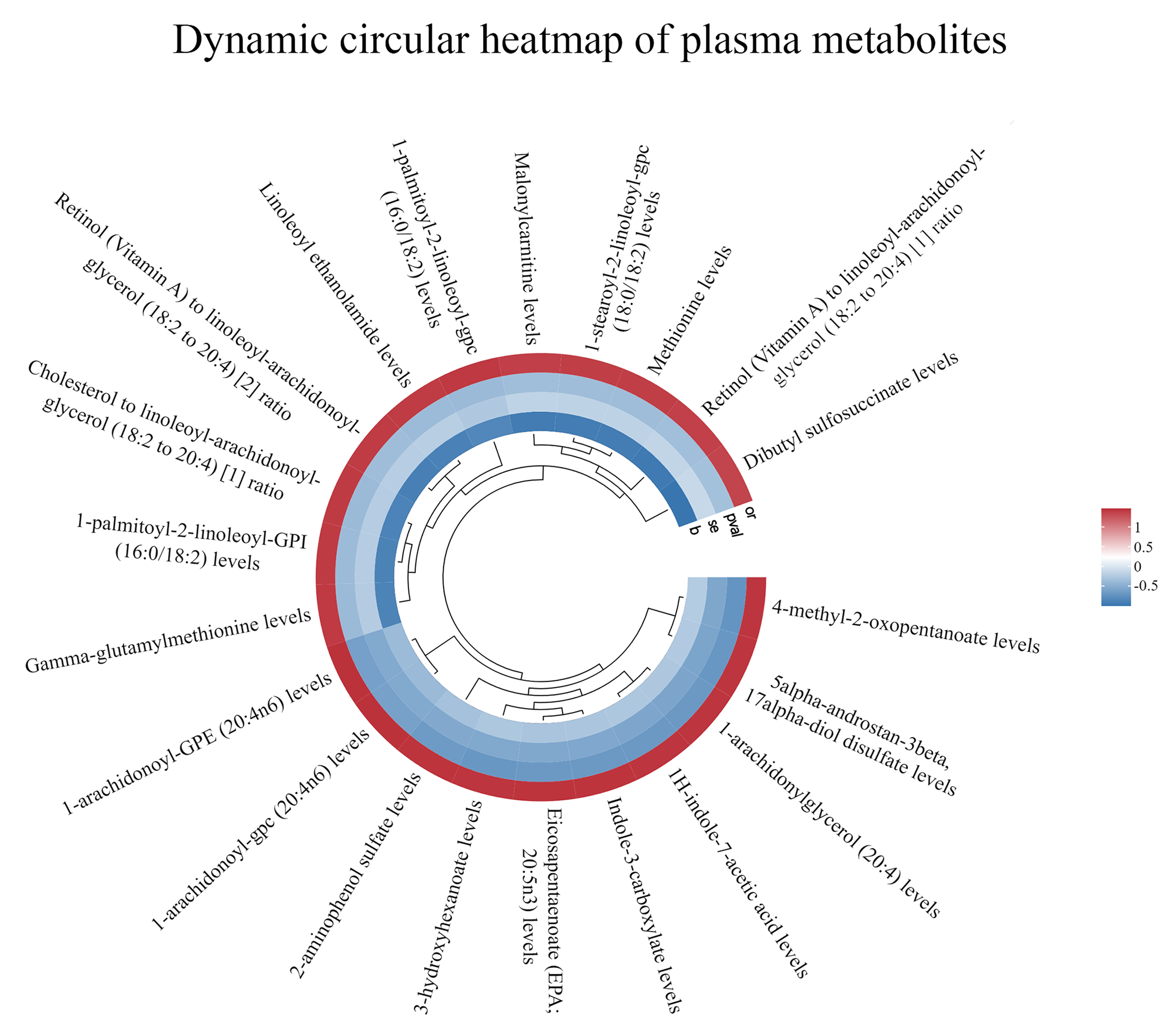

Supplement: Supplementary file 2 — Supplementary figures only (higher quality). [file jcav15p6698s2.zip › S-Figures/Figure S3.tif]

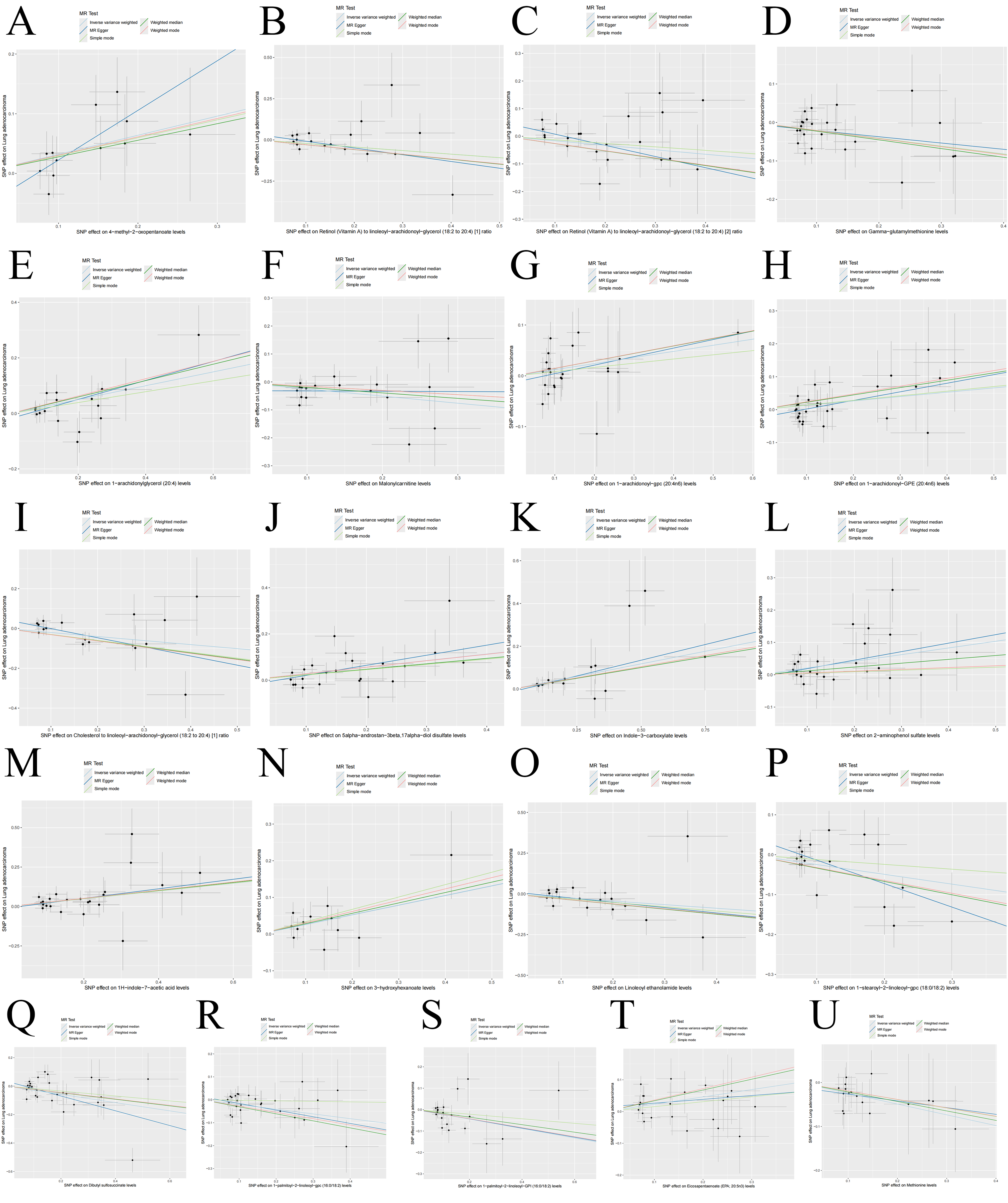

Supplement: Supplementary file 2 — Supplementary figures only (higher quality). [file jcav15p6698s2.zip › S-Figures/Figure S4.tif]

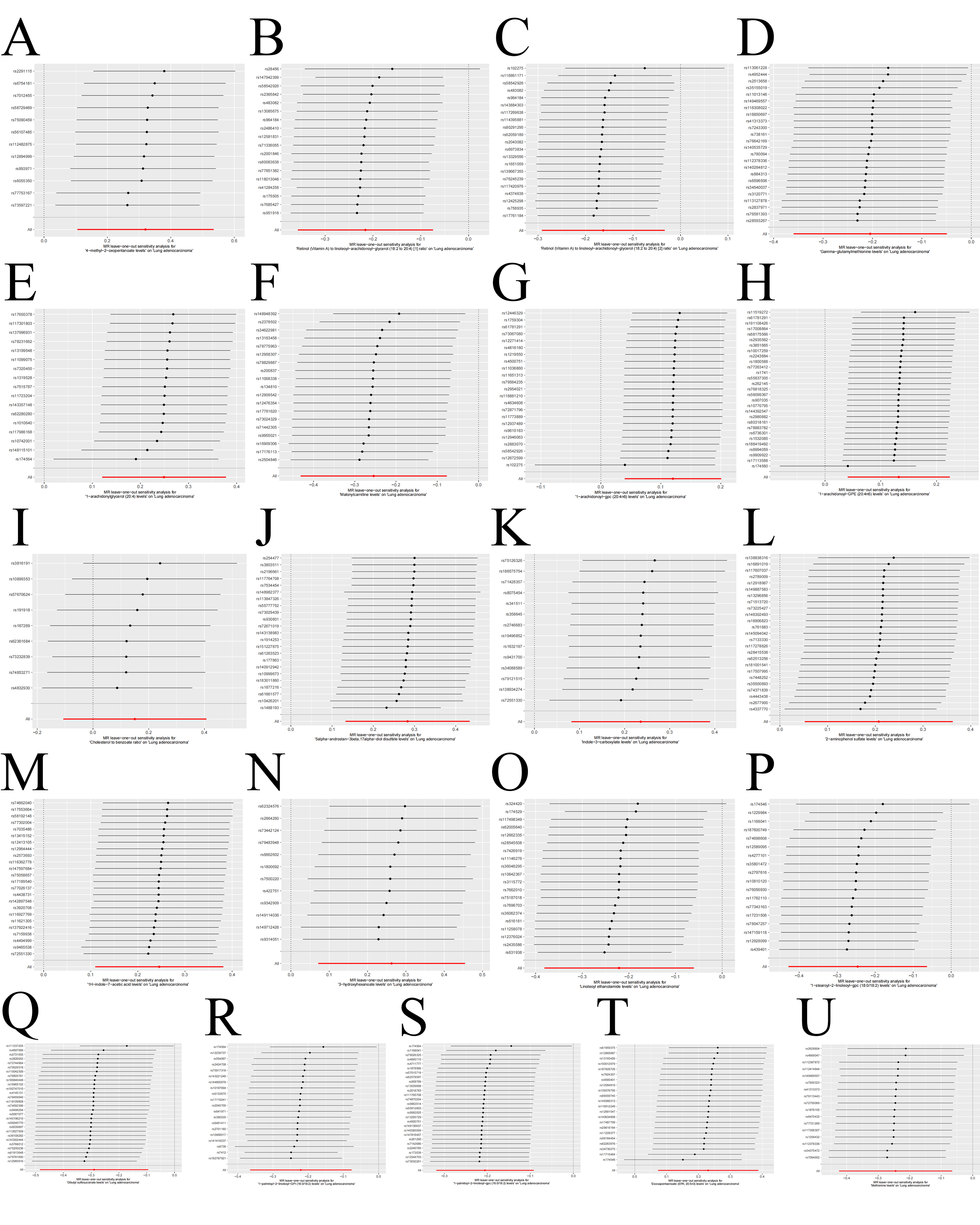

Supplement: Supplementary file 2 — Supplementary figures only (higher quality). [file jcav15p6698s2.zip › S-Figures/Figure S5.tif]

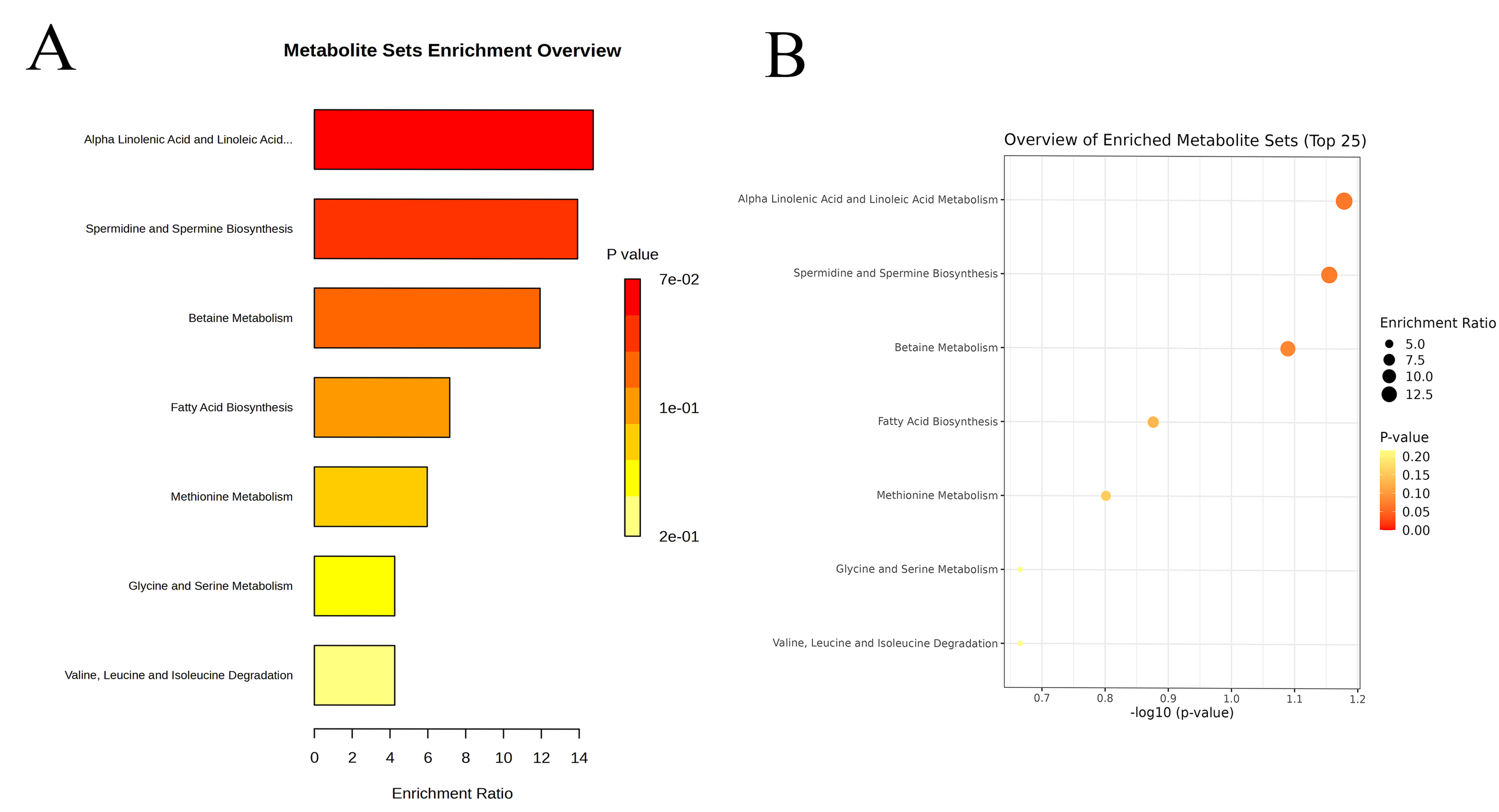

Supplement: Supplementary file 2 — Supplementary figures only (higher quality). [file jcav15p6698s2.zip › S-Figures/Figure S6.tif]

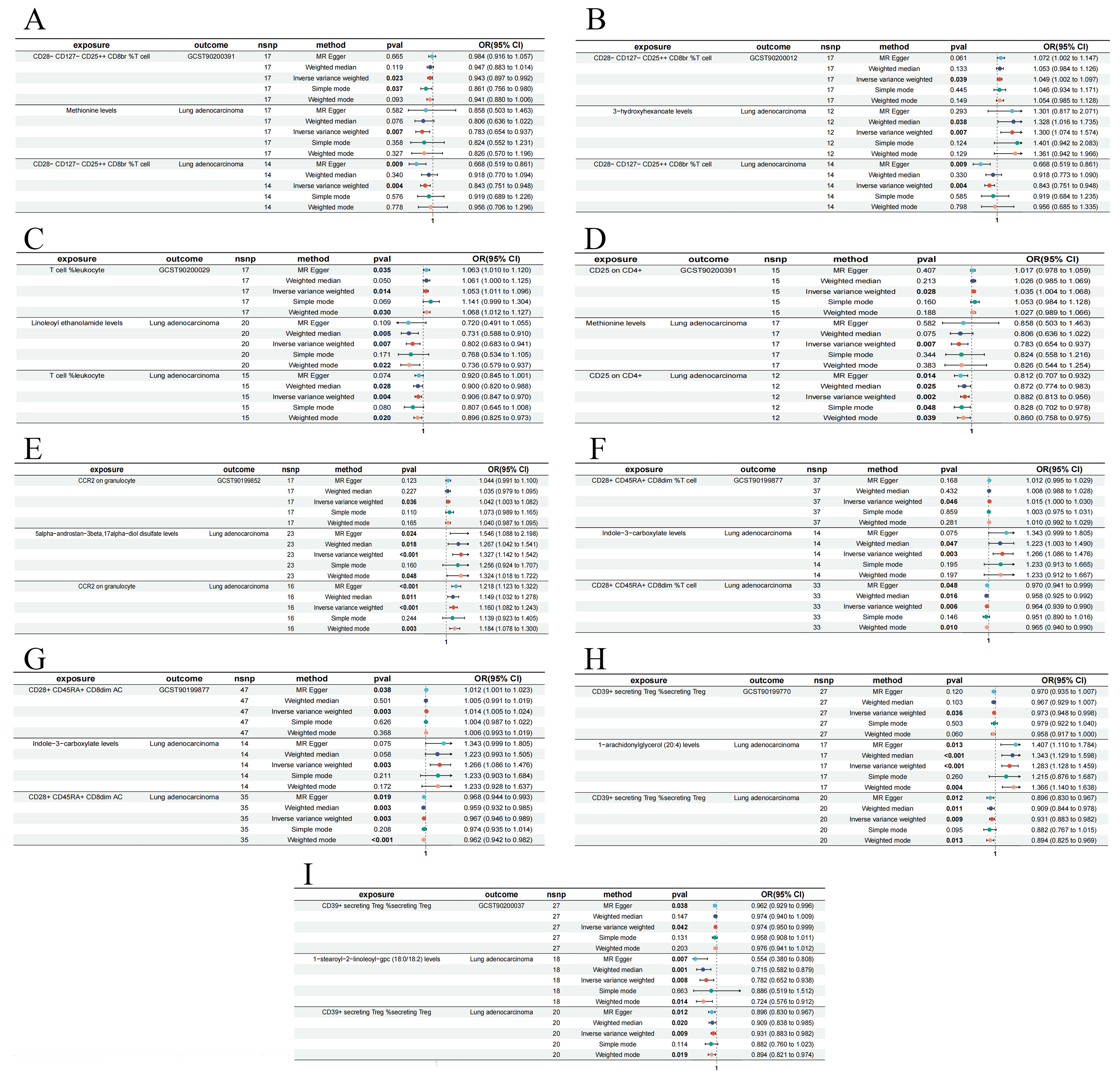

Supplement: Supplementary file 2 — Supplementary figures only (higher quality). [file jcav15p6698s2.zip › S-Figures/Figure S7.tif]
